# Japanese Classification of Esophageal Cancer, 12th Edition: Part I

**DOI:** 10.1007/s10388-024-01054-y

**Published:** 2024-04-03

**Authors:** Shinji Mine, Koji Tanaka, Hiroshi Kawachi, Yasuhiro Shirakawa, Yuko Kitagawa, Yasushi Toh, Takushi Yasuda, Masayuki Watanabe, Takashi Kamei, Tsuneo Oyama, Yasuyuki Seto, Kentaro Murakami, Tomio Arai, Manabu Muto, Yuichiro Doki

**Affiliations:** 1 Department of Esophageal and Gastroenterological Surgery, Juntendo University Hospital, 3-1-3, Hongo, Bunkyo-Ku, Tokyo, Japan; 2 Department of Gastroenterological Surgery, Graduate School of Medicine, Osaka University, Osaka, Japan; 3 Division of Pathology, Cancer Institute, Japanese Foundation for Cancer Research, Tokyo, Japan; 4 Department of Surgery, Hiroshima City Hiroshima Citizens Hospital, Hiroshima, Japan; 5 Department of Surgery, Keio University School of Medicine, Tokyo, Japan; 6 Department of Gastroenterological Surgery, National Hospital Organization Kyushu Cancer Center, Fukuoka, Japan; 7 Department of Surgery, Faculty of Medicine, Kindai University, Osaka, Japan; 8 Department of Gastroenterological Surgery, Cancer Institute Hospital of Japanese Foundation for Cancer Research, Tokyo, Japan; 9 Department of Surgery, Tohoku University Graduate School of Medicine, Miyagi, Japan; 10 Department of Endoscopy, Saku Central Hospital Advanced Care Center, Nagano, Japan; 11 Department of Gastrointestinal Surgery, Graduate School of Medicine, The University of Tokyo, Tokyo, Japan; 12 Department of Frontier Surgery, Graduate School of Medicine, Chiba University, Chiba, Japan; 13 Department of Pathology, Tokyo Metropolitan Institute for Geriatrics and Gerontology, Tokyo, Japan; 14 Department of Clinical Oncology, Kyoto University Hospital, Kyoto, Japan

**Keywords:** Esophageal cancer, Japanese classification, Endoscopic treatment, Surgery, Chemotherapy, Radiotherapy

## Abstract

This is the first half of English edition of Japanese Classification of Esophageal Cancer, 12th Edition that was published by the Japan Esophageal Society in 2022.

**Contents**

- Preface
- General principles of the 12th edition
- Abbrevi ation
- Terminology of the lymph nodes
- General rules

**General rules**

- 1. Purpose, object, and methods of descriptions
  - 1.1.　Purpose
  - 1.2.　Object
  - 1.3.　Methods of descriptions
    - 1.3.1. Principles of descriptions and abbreviations
- 2. Description of primary tumor
  - 2.1.　Number of primary tumors, size, and circumferential location
  - 2.2.　Tumor location
    - 2.2.1.　Anatomical definition of esophagus
    - 2.2.2.　Anatomical subsites of esophagus
    - 2.2.3.　Principles of description of tumor location
  - 2.3. Macroscopic tumor type
    - 2.3.1. Principles of macroscopic tumor type
    - 2.3.2. Macroscopic tumor type
    - 2.3.3. Subclassification of superficial type (type 0)
  - 2.4. Depth of tumor invasion (T)
    - 2.4.1.　Classification of depth of tumor invasion (T)
    - 2.4.2.　Diagnostic criteria for depth of superficial esophageal cancer by magnifying endoscopy
    - 2.4.3.　Diagnosis of cT4 invasion of adjacent organs by CT and other imaging techniques
      - 2.4.3.1. Typical CT images: cT3r
      - 2.4.3.2. Typical CT images: cT3br
      - 2.4.3.3. Typical CT images: cT4
  - 2.5. Infiltrative growth pattern (INF)
  - 2.6. Lymphatic or venous invasion
    - 2.6.1.　Lymphatic invasion (Ly)
    - 2.6.2.　Venous invasion (V)
  - 2.7. Description of surgical findings
    - 2.7.1.　Tumor size
    - 2.7.2. Distance from surgical margin to the tumor
    - 2.7.3. Surgical tumor type
    - 2.7.4. Surgical margin
      - 2.7.4.1. Proximal margin (PM)
      - 2.7.4.2. Distal margin (DM)
      - 2.7.4.3. Radial margin (RM)
  - 2.8.　Description of endoscopic findings
    - 2.8.1. Number of tumors and resected specimens
    - 2.8.2. Size of resected specimen and size of each tumor lesion
    - 2.8.3. Tumor types
    - 2.8.4. Resection margin
      - 2.8.4.1. Horizontal margin (HM)
      - 2.8.4.2.　Vertical margin (VM)
  - 2.9. Multiple primary cancers
    - 2.9.1. Multiple primary cancers of the esophagus
    - 2.9.2. Multi-organ primary cancers including the esophagus
  - 2.10. Intramural metastasis (IM)
- 3. Description of lymph nodes
  - 3.1.　Name, number, and extent and boundaries of lymph node station in esophageal cancer
  - 3.2.　Regional lymph nodes
  - 3.3.　Grading of lymph node metastasis (N)
- 4. Distant organ and lymph node metastasis (M)
- 5. Stage
  - 5.1. Staging of cervical and thoracic esophageal cancer, and squamous cell carcinoma of the esophagogastric junction
  - 5.2. Staging of adenocarcinoma of the esophagogastric junction
  - 5.3. Degree of lymph node dissection and residual tumor
    - 5.3.1. Lymph node dissection
    - 5.3.2. Description of lymph node dissection
    - 5.3.3. Definition of extent of lymph node dissection (D)
    - 5.3.4. Residual tumor after endoscopic and surgical resection (R)
- 6. Handling of endoscopically and surgically resected specimen
  - 6.1. Handling of endoscopically resected specimen
  - 6.2. Handling of surgically resected specimen (primary tumor)
  - 6.3. Sectioning for resected specimen
    - 6.3.1. Preparation of resected specimens for sectioning
    - 6.3.2. Rule of sectioning for endoscopically resected specimen
    - 6.3.3. Rule of sectioning for surgically resected specimen

**Preface**

The 12th edition of the Japanese Classification of Esophageal Cancer was published seven years after the publication of the 11th edition in 2015 [1, 2].

Considering the increasing importance of preoperative treatment, we add and revise the descriptions of preoperative diagnosis, especially T4 and lymph node metastasis diagnoses, which are considered to have a significant impact on the treatment policy, and modify the Response evaluation criteria and also to ensure consistency with the Japanese Cancer Staging Manual, particularly for cancers of the esophagogastric junction. Consistency with the TNM classification of the Union for International Cancer Control (UICC) was aimed as much as possible in the previous revision. However, consistency with the N classification was omitted because our N classification had been based on the site relative to the main tumor and because there had been a complete difference in the view of supraclavicular lymph node metastasis. This new version adopts the classification based on the number of metastases consistent with the TNM classification [3]. Furthermore, by examining both the dissection efficacy index and recurrence frequency, the regional lymph nodes are formulated to be more consistent with actual clinical practice. In accordance with these changes in the staging of lymph node metastasis, we develop a new staging classification system in an era when preoperative treatment has become the standard using prognostic information from the National Comprehensive Registry of Esophageal Cancer.

The diagnostic criteria for esophagogastric junction cancer were determined, and a booklet consisting of seven pages was added in the previous edition. In this revision, the definition and description of esophagogastric junction cancer are jointly developed by the Japanese Society of Gastric Cancer. In addition, the regional lymph nodes for esophagogastric junction cancer are established, and a description of staging is added. Many discussions were conducted among the committee members that led to this revision. Although there are still some points to be discussed, we appreciate the considerable efforts made by the individual committee members.

September 2022.

**General principles of the 12th edition**

1. “Ae” is eliminated as a subsite of the esophagus, and “Jz” is newly defined as the esophagogastric junction zone. “Jz” is equal to the esophagogastric junction area in Nishi’s classification.
2. We sub-classify cT3 as resectable (cT3 resectable: cT3r) or borderline resectable (cT3 borderline resectable: cT3br) because of the difficulty in determining adjacent organ involvement on imaging. Reference CT images and supporting findings are added to the diagnoses of cT3r, cT3br, and cT4.
3. For the diagnosis of cN, recommended cut-off values are provided, referring to the diagnostic accuracy of lymph node metastasis by size in CT diagnosis. Diagnoses using PET are also included as a reference.
4. To classify the degree of lymph node metastasis, a classification based on the number of metastatic regional lymph nodes is adopted for consistency with the TNM classification. We then abandon the lymph node grouping related to tumor location in thoracic esophageal cancer and newly establish the common regional lymph nodes for thoracic esophageal cancer. Supraclavicular lymph nodes are classified as distant rather than regional. However, it is defined as M1a to distinguish it from other distant metastases (M1b), because dissection efficacy can be expected in some cases. Although 106pre, 106tbR, and 112aoP are often dissected in metastasis-positive cases, they are defined as distant metastasis M1b in this edition because of insufficient data on the dissection efficacy index.
5. The degree of lymph node dissection (D) is determined based on the pattern and extent of lymph node dissection in each type of esophagectomy. For thoracic esophageal cancer, D1: dissection less than D2; D2: two-field lymph node dissection; and D3: D2 + cervical lymph node dissection. We included 106tbL, 111, 8a, and 11p in the standard D2 dissection range but could be omitted.
6. With the prevalence of standard preoperative treatment for locally advanced esophageal cancer, the staging classification is divided into clinical and pathological staging classifications based on the latest data.
7. We jointly establish a definition and description of esophagogastric junction cancer with the Japanese Gastric Cancer Association and organize its management in the nationwide registry of gastric and esophageal cancers to facilitate further surveys in the future.
8. Although primary esophageal cancer is a non-target lesion in the RECIST criteria for determining the treatment efficacy in RECIST [4], its prognostic impact is significant; therefore, we develop an index for determining the efficacy of CT for primary lesions. Simultaneously, the criteria for determining the treatment efficacy of primary tumors using endoscopy are updated. In addition, the diagnostic criteria for local recurrence after CR, as judged by endoscopy, are established.
9. In line with advances in endoscopic diagnosis and treatment, the classification content (September 2012) established by the Japanese Esophageal Association's Committee for Diagnostic Criteria of Superficial Esophageal Cancer Depth by Enlarged Endoscopy is described in the reference terms[5].
10. Squamous intraepithelial neoplasia is reevaluated and revised [5, 6].
11. Previously existing surgical findings [s] (gross surgical findings, intraoperative imaging findings, and gross findings of resection specimens) and findings in the endoscopic treatment [e] (intraoperative and gross findings of resection specimens) are included in the final analysis and deleted from the list.

**Abbrevi ations**

A: Anterior
AD: Adventitia
AI: Invasion to the adjacent organs
APC: Argon plasma coagulation
BA: Brownish area
BLI: Blue laser imaging
Br: Borderline resectable
c: Clinical findings
Ce: Cervical esophagus
CR: Complete response
CRT: Chemoradiotherapy
CT: Chemotherapy
CTV: Clinical target volume
D: Extent of lymph node dissection
DM: Distal margin
DMM: Deep muscularis mucosae
E: Esophagus
EGJ: Esophagogastric junction
EI: Esophageal invasion
EMR: Endoscopic mucosal resection
EP: Epithelium
ER: Endoscopic resection
ESD: Endoscopic submucosal dissection
f: Final findings
G: Stomach
GI: Gastric invasion
GIST: Gastrointestinal stromal tumor
HH: Hiatus hernia
HM: Horizontal margin
HT: Hyperthermia
IM: Intramural metastasis
INF: Infiltrative growth pattern
IO: Immuno-oncology drug
Jz: Zone of esophagogastric junction
Laser: Laser therapy
LPM: Lamina propria mucosae
LR: Local recurrence
LSBE: Long-segment Barrett’s esophagus
Lt: Lower thoracic esophagus
Ly: Lymphatic invasion
Ly/V: Lymphatic invasion or venous invasion
M: Distant organ metastasis
MCT: Microwave coagulation therapy
MM: Muscularis mucosae
MP: Muscularis propria
Mt: Middle thoracic esophagus
N: Grading of lymph node metastasis
NBI: Narrow band imaging
p: Pathological findings
P: Posterior
PD: Progressive disease
PDT: Photodynamic therapy
Ph: Pharynx
PM: Proximal margin
PR: Partial response
R: Residual tumor
RECIST: Response evaluation criteria in solid tumors
RM: Radial margin
RR: Remarkable response
RT: Radiotherapy
SCE: Specialized columnar epithelium
SCJ: Squamocolumnar junction
SD: Stable disease
SIN: Squamous intraepithelial neoplasia
SM: Submucosal layer
SMM: Superficial muscularis mucosae
SSBE: Short-segment Barrett’s esophagus
T: Depth of tumor invasion
Te: Thoracic esophagus
Tis: Carcinoma in situ
Ut: Upper thoracic esophagus
V: Venous invasion
VM: Vertical margin
X: Cannot be assessed

**Terminology of the lymph nodes**

R: Right
L: Left
sm: Submandibular
spf: Superficial
ac: Accessory
tr: Tracheal
up: Upper
mid: Middle
rec: Recurrent nerve
tb: Tracheobronchial
pre: Pretracheal
ao: Paraaortic
pul: Pulmonary ligament

**General rules**

## 1. Purpose, object, and methods of descriptions

### 1.1. Purpose

“The Guidelines for Clinical and Pathological Studies on Carcinoma of the Esophagus was originally published in 1969 by the Japanese Society for Esophageal Diseases. Since then, the Society changed its name to the Japan Esophageal Society in 2003 and published the “Japanese Classification of Esophageal Cancer” in the Japanese language with some revisions to treatment results to keep up to date and provide a standard nomenclature. The Society is publishing a handbook in English language, entitled “The Japanese Classification of Esophageal Cancer” to promote the international use of the Guidelines and Classification.

### 1.2. Object

The term esophageal cancer in the Japanese Classification refers to cancer originating in the esophagus, and cancers metastatic to the esophagus are excluded. All primary malignant tumors of the esophagus should be described according to the Japanese Classification.

### 1.3. Methods of descriptions

#### 1.3.1. Principles of descriptions and abbreviations

Findings are recorded using the uppercase letters T (depth of tumor invasion), N (lymph node metastasis), and M (distant organ metastasis). The extent of each finding is expressed by Arabic numerals following each uppercase letter. “X” is used in unknown cases. Three categories of findings, namely Clinical, Pathological, and Final findings, are identified using the lowercase “c,” “p,” and “f,” respectively, before each uppercase letter. The “f” of Final findings can be omitted (Tables 1, 2). The checklists describing the Japanese Classification of Esophageal Cancer are shown in Tables 3 and 4.

**Table 1:** Principles of description

Clinical findings (c)	Pathological findings (p)	Final findings (f)^Note^
• Physical examination • Diagnostic imaging: X-ray, Endoscopy (including NBI magnification, Iodine staining, EUS), CT, MRI, PET • Biopsy and Cytology • Biochemical and Biological examination • Others (including Genetic studies)	• Pathological examination of materials obtained by surgical or endoscopic section resection • Frozen section diagnosis	• Comprehensive findings based on clinical, surgical, endoscopical, and pathological findings

*Note:* Surgical findings [s] (gross surgical, intraoperative imaging, and gross findings of resection specimens) and findings in the endoscopic treatment [e] (intraoperative and gross findings of resection specimens) that were previously present are now included in the final findings and deleted from the list

**Table 2:** Description methods

	Clinical findings	Pathological findings	Final findings
Depth of tumor invasion	cT	pT	(f) T
Lymph node metastasis	cN	pN	(f) N
Distant organ metastasis	cM	pM	(f) M
Intramural metastasis	cIM	pIM	(f) IM
Stage	cStage	pStage	(f) Stage
Proximal margin	–	pPM	(f) PM
Distal margin	–	pDM	(f) DM
Radial margin	–	pRM	(f) RM
Horizontal margin (EMR/ESD)	–	pHM	(f) HM
Vertical margin (EMR/ESD)	–	pVM	(f) VM
Residual tumor	–	pR	(f) R

*Note:* Findings modified by treatment methods other than surgery are abbreviated as follows

Abbreviations: *EMR* endoscopic mucosal resection, *ESD* endoscopic submucosal dissection

**Table 3:** Checklist for descriptions of the Japanese Classification of Esophageal Cancer (Surgically resected cases)

Tumor location: Ce, Ut, Mt, Lt, Jz
Size: Maximum length (mm) and orthogonally oriented maximum width (mm)
Macroscopic tumor type: Type 0–Ip, Type 0–Is, Type 0–IIa, Type 0–IIb, Type 0–IIc, Type 0–III, Type 1, Type 2, Type 3, Type 4, Type 5, Combined types, and others
Histological type: squamous cell carcinoma, basaloid squamous carcinoma, carcinosarcoma, adenocarcinoma, Barrett’s adenocarcinoma, adenosquamous carcinoma, mucoepidermoid carcinoma, adenoid cystic carcinoma, neuroendocrine neoplasma (neuroendocrine carcinoma and neuroendocrine tumor), undifferentiated carcinoma, other carcinoma, smooth muscle tumor, gastrointestinal stromal tumor (GIST), neurogenic tumor, lymphoid tumor, malignant melanoma, and others
Depth of tumor invasion: pTX, pT0, pT1a (EP, LPM, and MM), pT1b (SM1, SM2, and SM3), pT2, pT3, pT4a, and pT4b
Pattern of infiltration: INFa, INFb, and INFc
Lymphatic invasion: LyX, Ly0, Ly1a, Ly1b, and Ly1c
Venous invasion: VX, V0, V1a, V1b, and V1c
Intramural metastasis: pIMX, pIM0, and pIM1
Involvement of resection margins
Proximal margin: pPMX, pPM0, and pPM1
Distal margin: pDMX, pDM0, and pDM1
Radial margin: pRMX, pRM0, and pRM1
Multiple primary cancers: absent, and present (number)
Lymph node metastasis: pNX, pN0, pN1, pN2, and pN3 number of positive nodes (number for lymph node stations with positive nodes)
Distant metastasis: MX, M0, M1a, and M1b
Residual tumor: RX, R0, R1, and R2
Histological response of chemotherapy and/or radiation: No data, Grade 0, Grade 1a, Grade 1b, Grade 2, and Grade 3^*Note*^

*Note*: Histological response of chemotherapy and/or radiation is described according to the “Criteria for histological response of chemotherapy and/or radiotherapy” (refer to 18 in Part II)

**Table 4:** Checklist for descriptions of the Japanese Classification of Esophageal Cancer (Endoscopically resected cases)

Tumor location: Ce, Ut, Mt, Lt, and Jz
Size of specimen: length (mm), width (mm)
Size of tumor: length (mm), width (mm)
Multiple lesions: present, and absent
Macroscopic tumor type: Type 0–Ip, Type 0–Is, Type 0–IIa, Type 0–IIb, Type 0–IIc, Type 0–III, Combined types, and others
Histological type: Squamous cell carcinoma, Basaloid squamous carcinoma, Carcinosarcoma, Adenocarcinoma, Barrett adenocarcinoma, Adenosquamous carcinoma, Mucoepidermoid carcinoma, Adenoid cystic carcinoma, Neuroendocrine neoplasmas (neuroendocrine carcinoma and neuroendocrine tumor), Undifferentiated carcinoma, other carcinoma, Smooth muscle tumor, Gastrointestinal stromal tumor (GIST), Neurogenic tumor, Lymphoid tumor, Malignant melanoma, and others
Depth of tumor invasion: pTX, pT0, pT1a (EP, LPM, MM), and T1b (SM1, SM2)
Pattern of infiltration: INFa, INFb, and INFc
Lymphatic invasion: LyX, Ly0, and Ly1
Venous invasion: VX, V0, and V1
Horizontal margin: pHMX, pHM0, and pHM1
Vertical margin: pVMX, pVM0, and pVM1
Residual tumor: RX, R0, R1, and R2

The order of clinical description is as follow: Tumor location (including the distance from the incisor), circumferential extent, tumor length, macroscopic tumor type, histological type (when identified), depth of tumor invasion, lymph node metastasis, distant organ metastasis, and stage, e.g., Mt (31–36 cm), 1/2 of circumference and on the anterior wall, 5 cm, type 2, moderately differentiated squamous cell carcinoma, cT3, cN2, cM0, cStage IIIB.

The order of pathological description is as follow: Tumor location, tumor length, macroscopic tumor type, histological type, depth of tumor invasion, infiltrative growth pattern, lymphatic invasion, venous invasion, intramural metastasis, involvement of resection margins (proximal, distal, and radial margins), multiple primary cancers, histological response of chemotherapy and/or radiation, lymph node metastasis, distant organ metastasis, and stage, e.g., Mt, 5 cm; type 2, moderately differentiated squamous cell carcinoma; pT3, INFa, Ly1a, V1a, pIM0, pPM0, pDM0, pRM0, multiple primary carcinomas (present, two lesions), CRT-Grade 2, pN1 (2/30), sM0, f Stage IIIA.

Other items to be filled in

1. Metastasis from other organs and cancer invasion
2. Non-cancerous coexisting tumors: leiomyoma and others
3. Other: Barrett's esophagus, achalasia, and other background lesions

## 2. Description of primary tumor

### 2.1. Number of primary tumors, size, and circumferential location

The maximum length (mm), orthogonally oriented maximum width (mm), the center of circumferential extent, and the proportion of the tumor in the entire circumference should be described. In addition, the diagnostic methods used, such as barium radiography, endoscopy, and EUS, should be recorded.

### 2.2. Tumor location

#### 2.2.1. Anatomical definition of esophagus

The esophagus is anatomically defined as extending from the esophageal orifice to the esophagogastric junction. The esophageal orifice is located at the lower margin of the cricoid cartilage. Identification of the esophagogastric junction (EGJ) is described in Part II (refer to 8.1 in Part II).

#### 2.2.2. Anatomical subsites of esophagus (Fig. 1)

**Fig. 1:**
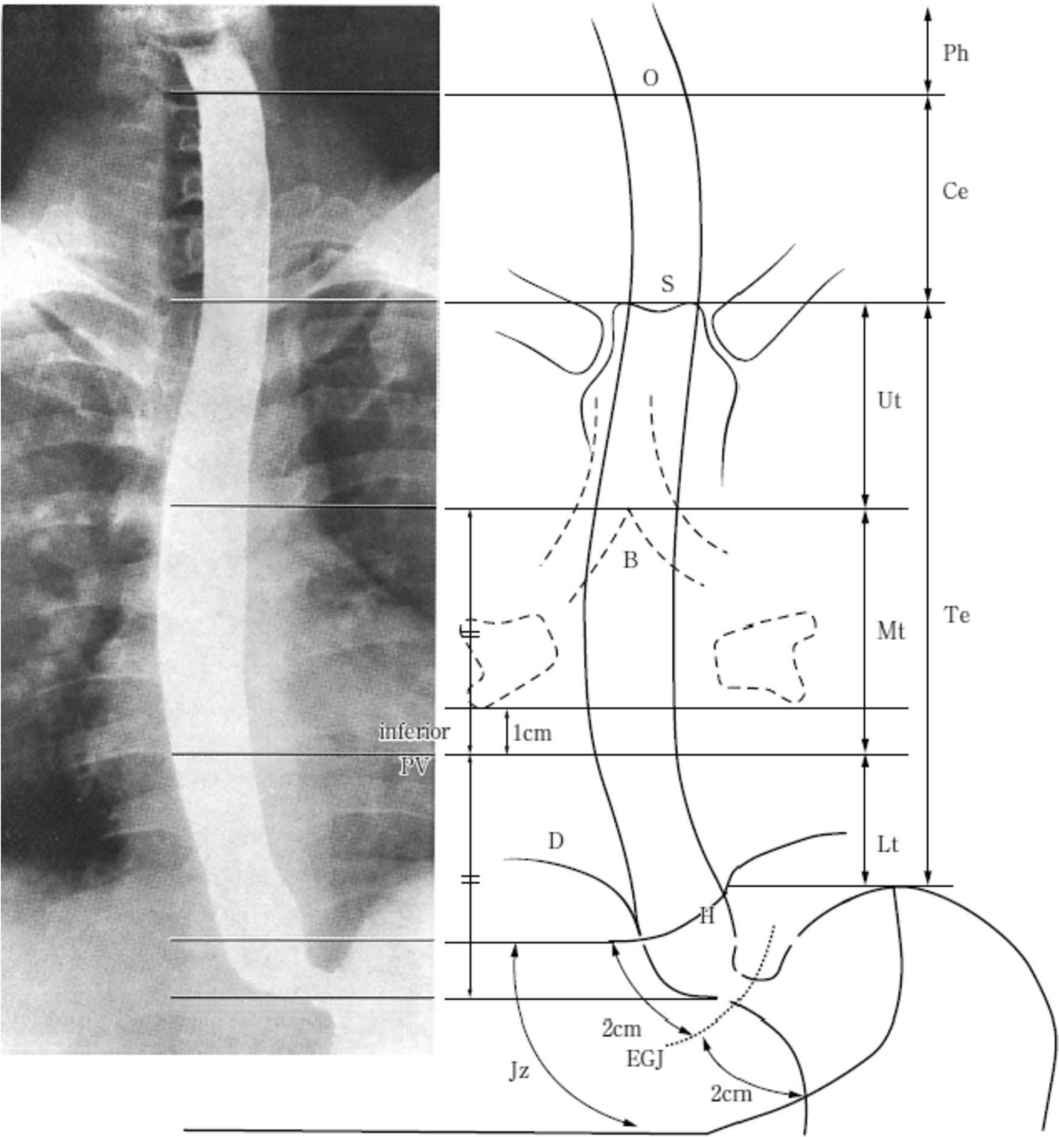
Tumor location. O: esophageal orifice, S: superior margin of the sternum, B: tracheal bifurcation, PV: pulmonary vein, D: diaphragm, EGJ: esophagogastric junction, H: esophageal hiatus

The esophagus lies between the hypopharynx and stomach and can be anatomically divided into the cervical esophagus (Ce), thoracic esophagus (Te), and the zone of the esophagogastric junction (Jz).

Cervical esophagus (Ce): From the esophageal orifice to the sternal notch.

Thoracic esophagus (Te): From the sternal notch to 2 cm cephalad of the esophagogastric junction.

Upper thoracic esophagus (Ut): From the sternal notch to the tracheal bifurcation

Middle thoracic esophagus (Mt): The proximal half of the two equal portions between the tracheal bifurcation and the esophagogastric junction

Lower thoracic esophagus (Lt): The portion between the inferior margin of the Mt and 2 cm cephalad from the esophagogastric junction

Zone of the esophagogastric junction (Jz): The zone of the esophagogastric junction is defined as the region 2 cm above the esophagogastric junction and 2 cm below the esophagogastric junction.

*Note 1*: In the absence of esophagography, the border between the Mt and Lt should be 1 cm distal to the inferior border of the inferior pulmonary vein on CT.

*Note 2*: When Jz shifts into the thoracic cavity due to a hiatal hernia of the esophagus, first, the Jz range should be decided based on the esophagogastric junction. And then, the remaining range is defined as Lt.

#### 2.2.3. Principles of description of tumor location

When the tumor location is uncertain because other examinations except endoscopy are yet to be performed, only the distance from the incisor is described.

When the tumor extends continuously into more than one portion of the esophagus, the main tumor location is the site of the deepest tumor invasion, which should be described first. When it is difficult to determine the site of the deepest tumor invasion, the portion at the central point of the tumor can be recorded as the main tumor location.

In the case of multiple primary lesions, their locations are described in the order of depth of tumor invasion. The deepest lesion is described first. When it is difficult to determine the order of the depth, then, the order of description depends on the size of the area occupied by the lesion. The largest lesion is described first, e.g., MtLt, LtJzG, CePh.

Esophagogastric junction cancer (refer to 8.4 in Part II)

1. The location of the lesion should be described depending on the tumor center as follows: E, EG, E = G, GE, or G.
2. The tumor center is recorded as the distance (− 2 cm, 1 cm, and others) from the esophagogastric junction. Minus means that the tumor center is located at the esophagus, and plus means that tumor center is located at the stomach.
3. The lengths of proximal and distal invasions are recorded as the distance from the EGJ (cm).
4. Barrett's esophagus, hiatal hernia, and other coexisting lesions are recorded.

### 2.3. Macroscopic tumor type

#### 2.3.1. Principles of macroscopic tumor type

Tumor type classification is based on macroscopic findings. Radiological findings and endoscopic findings are also defined according to macroscopic findings.

Tumors confined to the mucosa or submucosa are classified as the superficial type, while tumors whose invasion extends to the muscularis propria or beyond are classified as the advanced type. The superficial types have the prefix ‘0’ and are classified into 0-I, 0-II, or 0-III. The advanced types are divided into four categories:1, 2, 3, or 4. When a tumor cannot be classified into any of these 5 (0–4) categories or consists of their combinations, it is classified as 5.

#### 2.3.2. Macroscopic tumor type (Figs. 2, 3, 4, 5, 6, 7)

**Fig. 2:**
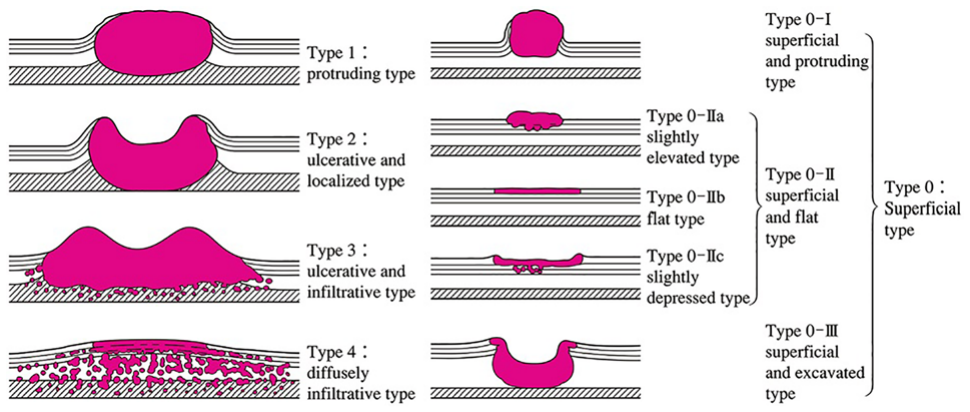
Macroscopic classification (Type 0–4)

**Fig. 3:**
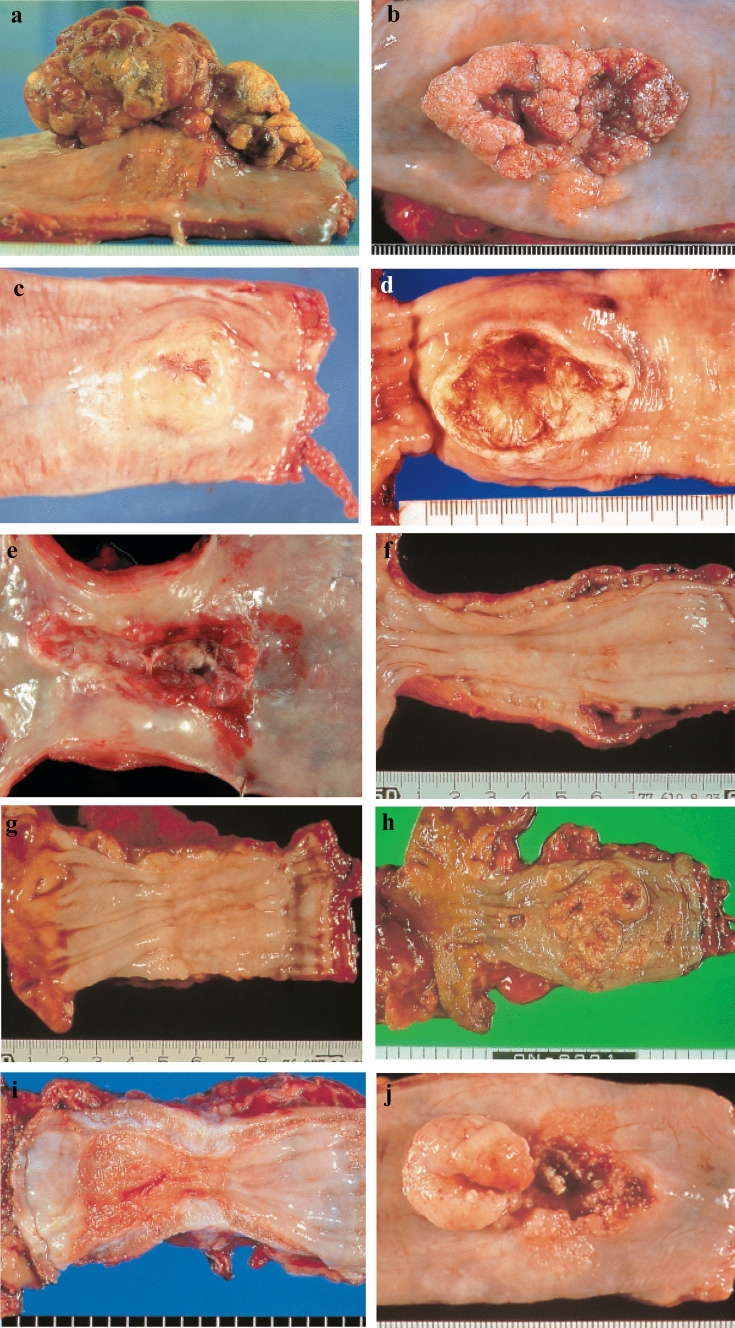
**a** Type 1: A pedunculated and tall polypoid lesion. This is judged to be advanced cancer based on its size, immobility (or cut cross section). **b** Type 1: This protruding lesion with a clearly demarcated border has lobules or a papillary appearance on its surface. **c** Type 1: Most of the surface of the protrusion is covered by non-cancerous epithelium. This is judged to be advanced cancer based on its size and immobility. **d** Type 2: This lesion is a deep ulcer with a well-demarcated surrounding ridge. **e** Type 3: This lesion is a deep ulcer surrounded by a poorly demarcated ridge. The lesion extends circumferentially causing luminal stenosis. **f** Type 4: This diffusely invasive lesion with no clear margin makes the esophageal wall thick and hard and causes luminal stenosis. No distinct ulcer can be seen. **g** Type 4: The thickening of the esophageal wall and the edematous changes of the mucosa suggest diffuse intramural extension of the lesion, but there is no finding of hardening or stenosis, and no finding of ulcer formation. **h** Type 5a: The macroscopic appearance is extremely complex with Type 1, and Type 2 and others, and it is difficult to categorize. **i** Type 5b: This macroscopic tumor (Type 5b) cannot be categorized because of preoperative chemoradiotherapy. **j** Combined Type. This cancer showed mixed morphology of advanced Type 1, Type 2, and 0-IIc.

**Fig. 4:**
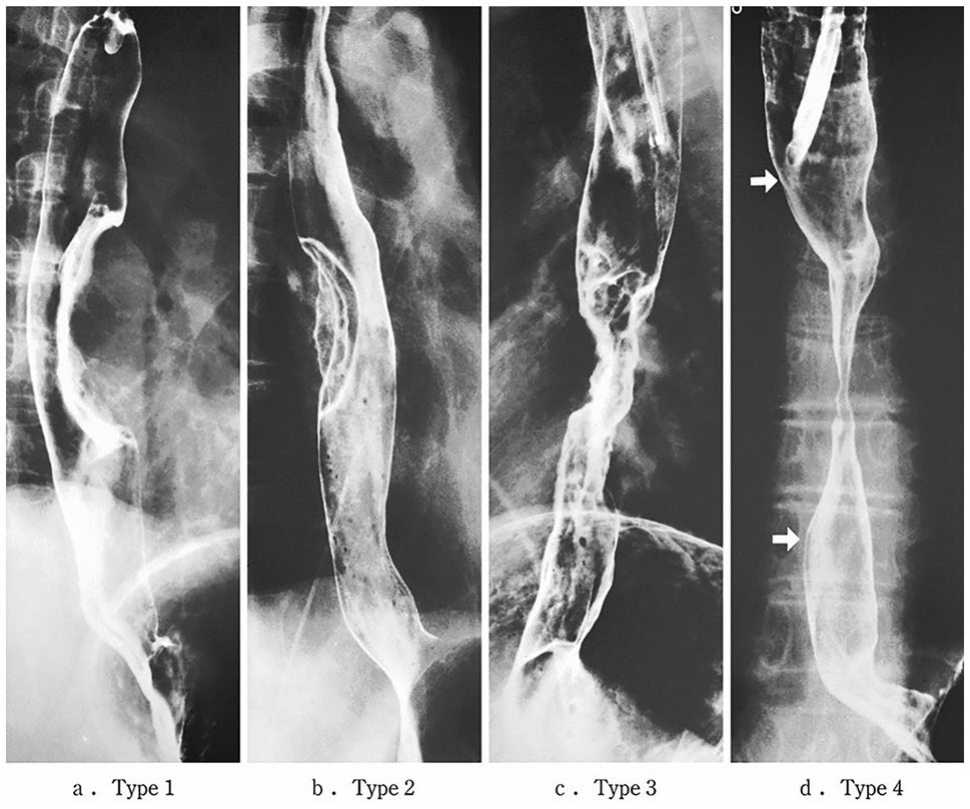
Roentgenological findings advanced type

**Fig. 5:**
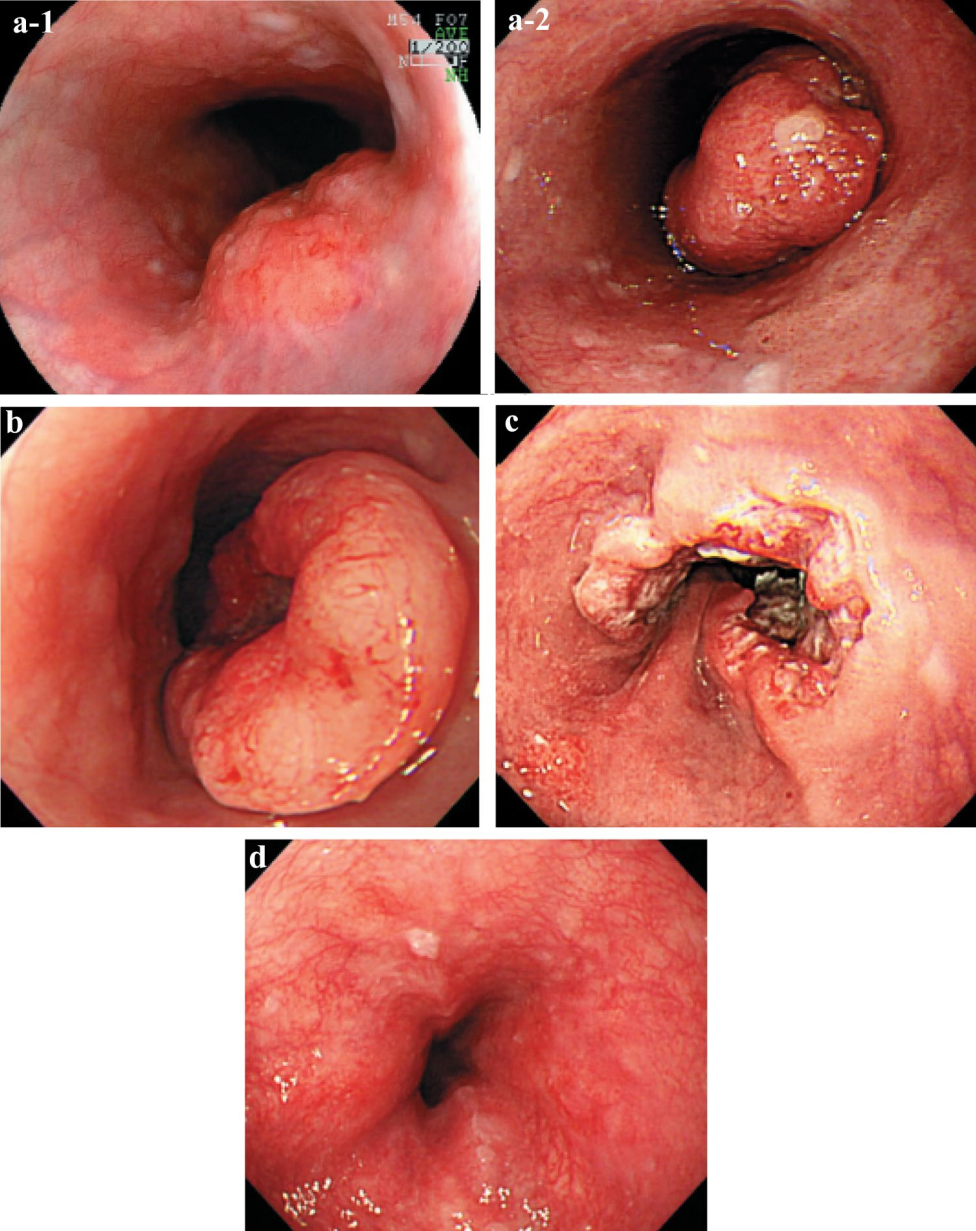
**a-1** Type 1, protruding type (pT2): A tall lesion with a broad base. **a-2** Type 1, protruding type (pT2): A tall lesion with a narrow base. **b** Type 2, ulcerative and localized type (pT3). A deep ulcerative lesion surrounded by a well-demarcated ridge. **c** Type 3, ulcerative and infiltrative type (pT3). A deep ulcerative lesion surrounded by an ill-demarcated ridge. **d** Type 4, diffusely infiltrative type (pT3). Ill-defined thickening and hardening of the esophageal wall accompanied by luminal stenosis is observed. There is no remarkable ulcer formation

**Fig. 6:**
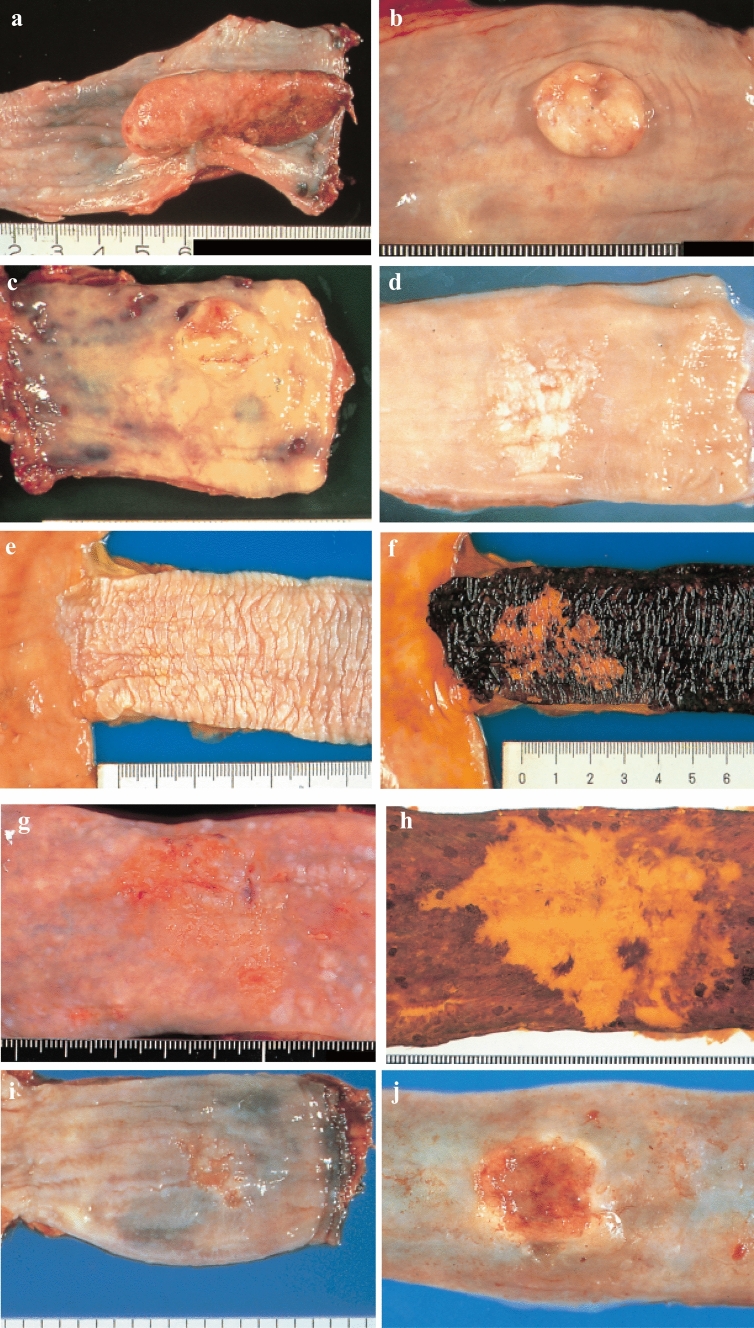

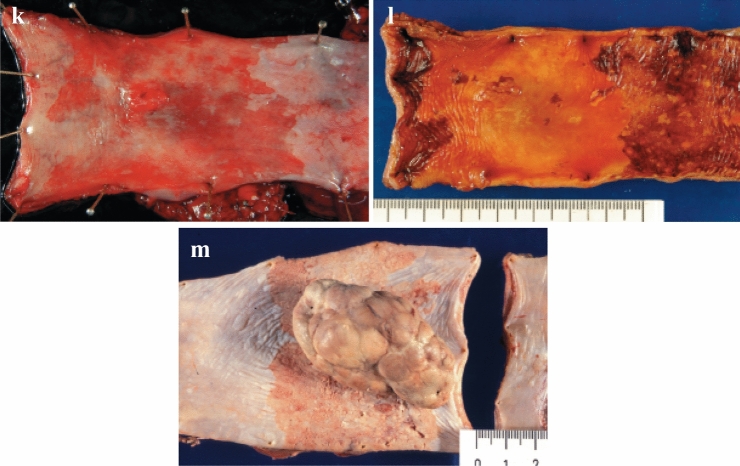
**a** Type 0-Ip (superficial, protruding type, and pedunculated). The tumor is well demarcated and has a narrow base. **b** Type 0-Ip (superficial, protruding type and pedunculated). The well-demarcated, protruding tumor has an irregular and nodular surface. **c** Type 0-Is (superficial protruding type, and sessile). The surface of this ill-demarcated tumor is mostly covered by the normal epithelium. **d** Type 0-IIa (slightly elevated type). The generally white tumor is only slightly elevated from the mucosa. **e** Type 0-IIb (Flat type). Only minute irregularities and no macroscopic abnormal features are observed. **f** Type 0-IIb (Flat type) (Iodine-stained view of **e**). The superficial tumor is unstained by iodine. **g** Type 0-IIc (slightly depressed type). The superficial depressed lesion has no clear margin and a finely granular surface. **h** Type 0-IIc (Iodine-stained view of **g**) The superficial tumor is unstained by iodine. **i** Type 0-IIc (slightly depressed type). The superficial depressed lesion has an irregular margin. **j** Type 0-III (superficial and depressed type). The deeply depressed lesion with a slightly elevated margin suggests invasion beyond the muscularis mucosa. **k** Type 0-IIc + “0-IIa” (superficial spreading type). The widespread slightly depressed red lesion (0-IIc) has a slightly elevated lesion (0-IIa) in its center, suggesting invasion into the submucosal layer. The lesion, more than 5 cm in length, is defined as the superficial spreading type. **l** Type 0-IIc + “0-IIa” (superficial spreading type) (Iodine-stained view of **k**). The reddish depressed lesion is not stained with iodine solution. **m** Type 0-IIc + “0-Ip”. The well-demarcated protruding tumor with a narrow base (0-Ip) has a slightly depressed lesion (0-IIc) in the surrounding area

**Fig. 7:**
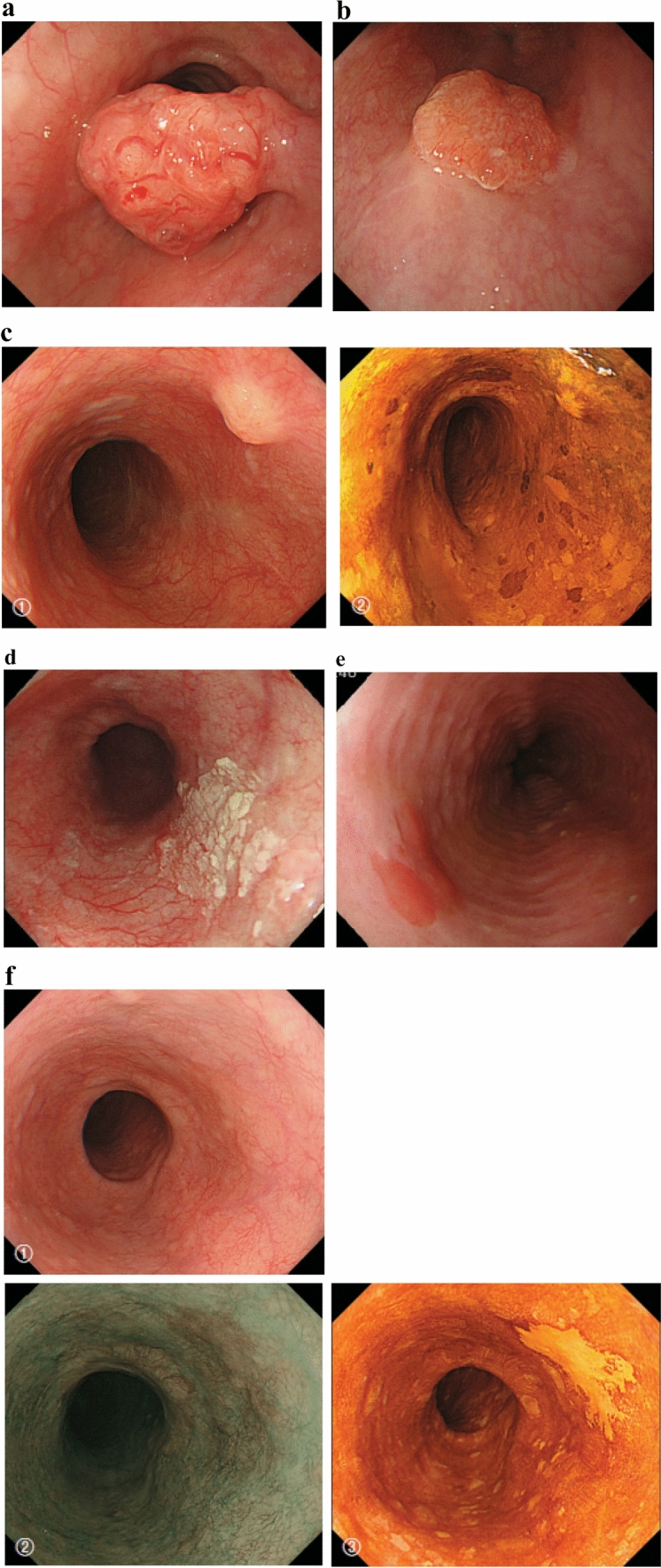

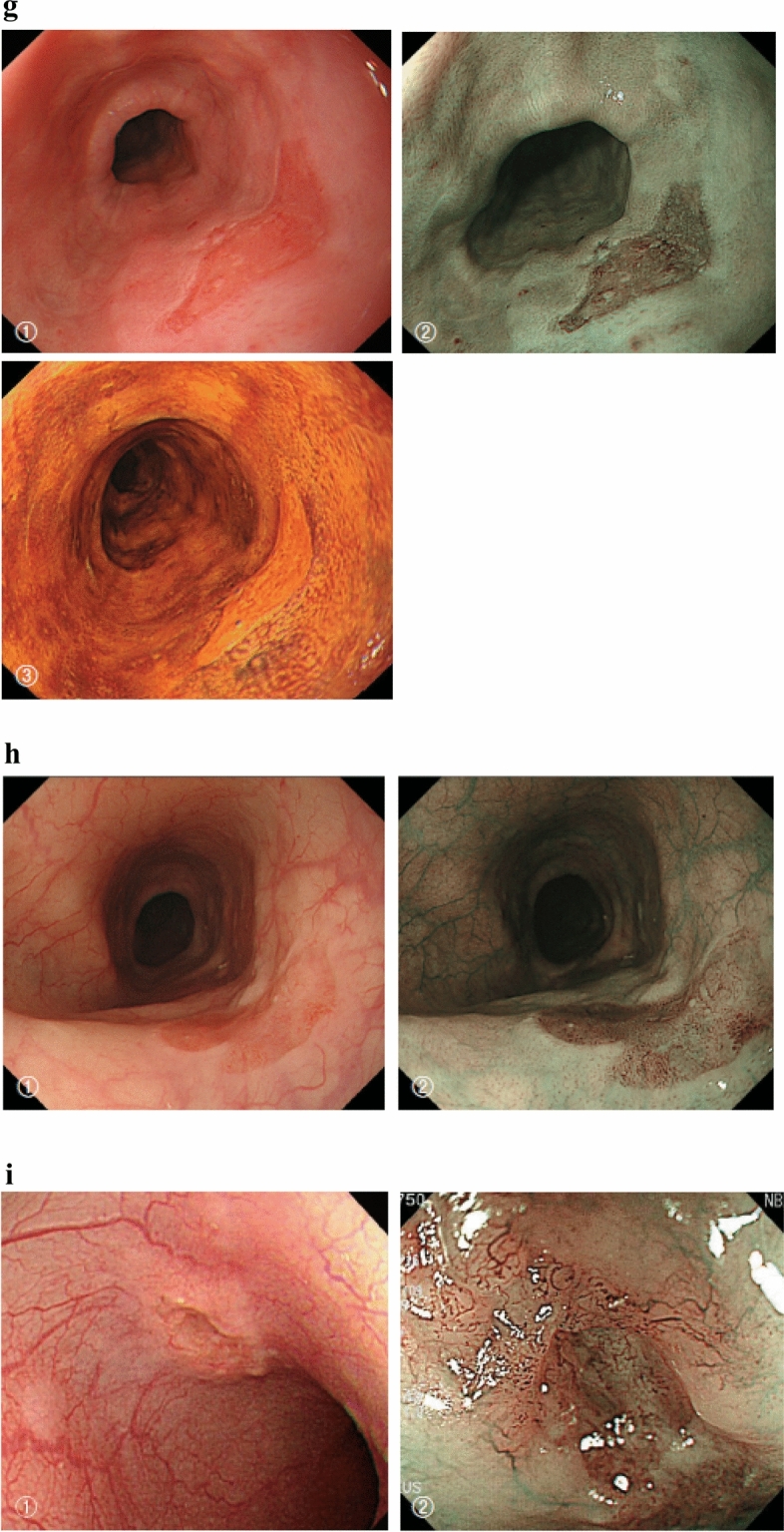

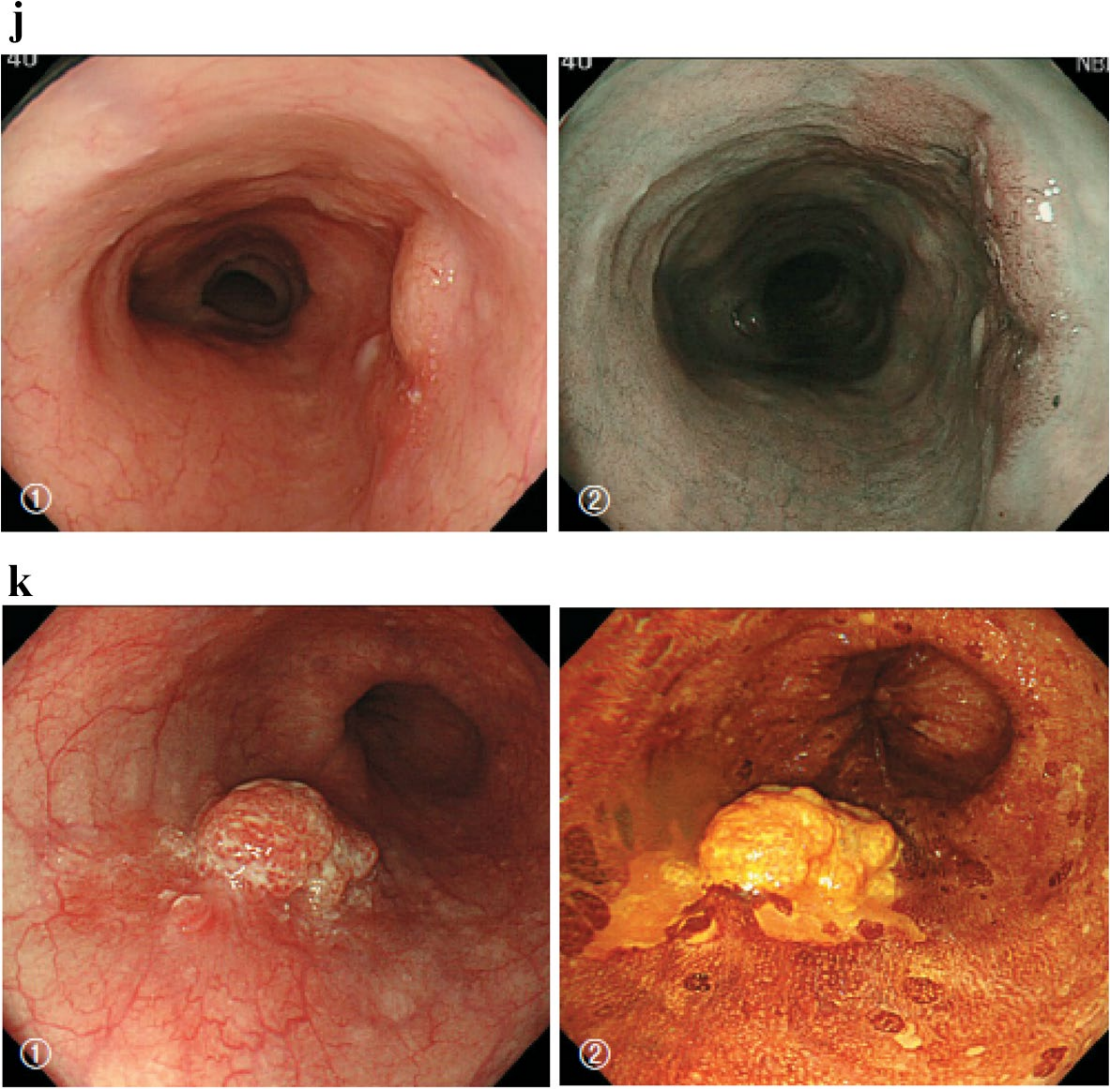
**a** Type 0-Ip, superficial, protruding type, and pedunculated (cT1b-SM2-3). A well-demarcated protruding and pedunculated tumor shows an irregular and nodular surface. **b** Type 0-Is, Superficial and protruding type, sessile (pT1b-SM2). A well-demarcated protruding and sessile tumor. **c** Type 0-Is, superficial, protruding type, and sessile (pT1b-SM2). 1 Conventional endoscopy: An ill-demarcated protruding tumor covered by normal esophageal mucosa suggests a tumor mass in the submucosa. 2 Iodine staining: The mucosa covering the tumor is stained brown, and an unstained area at the top suggests exposed tumor tissue. **d** Type 0-IIa, slightly elevated type (pT1a-MM). A plaque-like, slightly elevated white tumor. Tumor invasion of the white area remains within the lamina propria, while a tiny protrusion at the distal margin of the tumor invades the muscularis mucosa. **e** Type 0-IIa, slightly elevated type (pT1a-EP). A slightly elevated tumor with well-demarcated reddening (the height of a type 0-IIa lesion is less than 1 mm). **f** Type 0-IIb flat type (pT1a-EP). 1 Conventional endoscopy: A conventional observation cannot detect the lesion. 2 Narrow band imaging: A brownish area can be detected. 3 Iodine staining: A completely flat lesion is identified as a well-demarcated, unstained area using iodine staining. **g** Type 0-IIc, slightly depressed type (pT1a-LPM). 1 Conventional endoscopy: An irregularly shaped mucosal reddening with a slight depression is visible. 2 Narrow band imaging: The lesion is also visible as a brownish area. 3 Iodine staining: A well-demarcated, unstained area is visible using iodine staining. **h** Type 0-IIc, slightly depressed type (pT1b-SM1). 1 Conventional endoscopy: An area of mucosal reddening with a slight depression and marginal elevation is visible. 2 Narrow band imaging: A brownish area suggesting a hypervascular lesion is visible. **i** Type 0-III, Superficial and excavated type (cT1b-SM2-3). 1 Conventional endoscopy: A distinctly depressed lesion with a surrounding elevated area is visible, suggesting an ulcer reaching the muscularis mucosa. 2 Narrow band imaging: A well-demarked lesion with surrounding elevated area is visible as a brownish area. **j** Combined type 0-IIc + ”0-Is” (pT1b-SM2). 1 Conventional endoscopy: A distinct elevation with a wider base is visible. A slightly depressed lesion close to the distal margin is also observed. 2 Narrow band imaging: A lesion with a well-demarcated margin is visible. **k** Combined type 0-Is + 0- IIc (pT1b-SM2). 1 Conventional endoscopy: A distinctly protruding lesion with a wide base and irregular nodular changes is visible. Reddening of the esophageal mucosa close to the lesion with an ill-defined margin is suspected. 2 Iodine staining: The margin of the mucosal change is identified as well-demarcated, unstained area

- Type 0: Superficial
- Type 1: Protruding
- Type 2: Ulcerative and localized
- Type 3: Ulcerative and infiltrative
- Type 4: Diffusely infiltrative
- Type 5: Unclassifiable
  - Type 5a: Unclassifiable without treatment
  - Type 5b: Unclassifiable after treatment ^*Notes 1, 2*^

*Note 1:* Macroscopic tumor type before any treatments is described.

*Note 2:* In case with preceding treatments, if the tumor still can be classified into Type 0–4, describe it, otherwise describe it as Type 5b. In either case, all the prefixes indicating the preceding treatments, e.g., CT-, RT-, EMR-, should be marked.

#### 2.3.3. Subclassification of superficial type (Type 0) (Figs. 6 and 7)

- Type 0-I: Superficial and protruding
  - Type 0-Ip: Pedunculated
    Type 0-Is: Sessile (broad based)
- Type 0-II: Superficial and flat
  - Type 0-IIa: Slightly elevated
    Type 0-IIb: Flat
    Type 0-IIc: Slightly depressed
- Type 0-III: Superficial and excavated
- Other notations

*Note 1*: Combined type: When multiple macroscopic tumor types are mixed in one lesion, it is called a combined type. The wider tumor is described first, and the smaller one is second. Double quotation marks (“”) are placed around the macroscopic tumor type that has the deepest tumor invasion. The main macroscopic tumor type was the deepest. However, when an advanced type is mixed, the most advanced type is described first and double quotation marks are unnecessary. For example, 0-IIc + “0-Is”, 3 + 0-IIc.

*Note 2*: Superficial spreading type: superficial type 0-IIc in which the maximal length of the tumor extends ≥ 5 cm longitudinally. This may also be described in macroscopic tumor types.

### 2.4. Depth of tumor invasion (T) (Fig. 8)

**Fig. 8:**
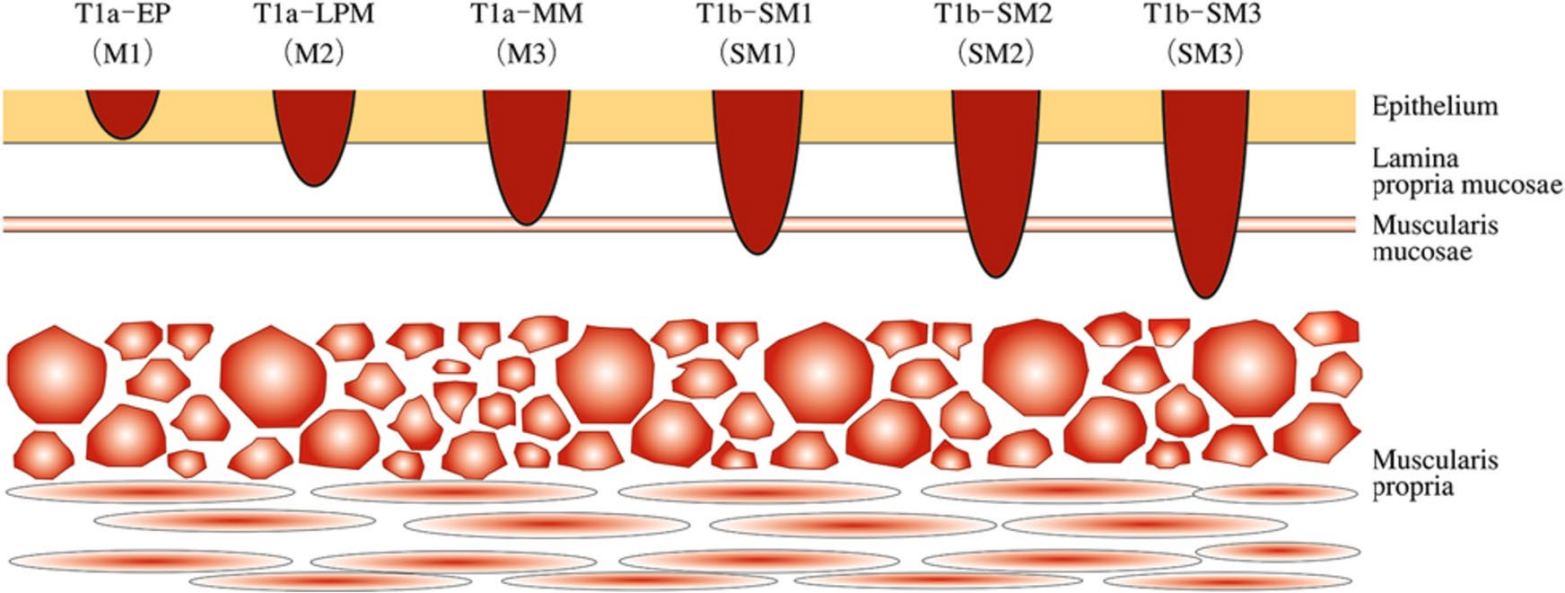
Subclassification for superficial cancer

#### 2.4.1. Classification of depth of tumor invasion (T)

- TX: Depth of tumor invasion cannot be assessed
- T0: No evidence of primary tumor
- T1a: Tumor invades mucosa^*Notes 1 and 2*^
  - T1a-EP: Carcinoma in situ (Tis)
    T1a-LPM: Tumor invades lamina propria mucosae (LPM)
    T1a-MM: Tumor invades muscularis mucosae (MM)
- T1b: Tumor invades submucosa (SM)^*Notes 1 and* 3^
  - T1b-SM1: Tumor invades the upper third of the submucosal layer
    T1b-SM2: Tumor invades the middle third of the submucosal layer
    T1b-SM3: Tumor invades the lower third of the submucosal layer
- T2: Tumor invades muscularis propria (MP)
- T3: Tumor invades adventitia (AD)^*Note 4*^
- T4: Tumor invades adjacent structures (AI)^*Notes 5, 6, and 7*^

*Note 1*: Superficial esophageal cancer: T1a and T1b are designated as superficial cancers, regardless of lymph node or distant organ metastases, e.g.*,* superficial esophageal cancer: T1NxMx.

*Note 2*: Early esophageal cancer: T1a can be designated as early esophageal cancer regardless of the presence or absence of lymph node or distant organ metastasis, e.g., early esophageal cancer: T1aNxMx.

*Note 3*: In endoscopically resected specimens of carcinoma other than adenocarcinoma, a tumor invading the submucosa to a depth of ≤200 μm from the muscularis mucosae is classified as T1b-SM1, while a tumor extending >200 μm is classified as T1b-SM2. Since the true entire thickness of the submucosal layer is unknown, SM3 is not defined.

In adenocarcinoma arising in the esophagus or esophagogastric junction, all lesions that invaded the submucosa >500 μm from the muscularis mucosae are considered T1b-SM2.

For pT1b carcinomas, the invasion distance from the lower edge of the muscularis mucosae is also measured. Invasion distance is described only in endoscopically resected specimens, e.g., pT1b-SM2 (600 μm).

*Note 4*: The T3 subclassification is used only for clinical diagnosis.

T3r: Resectable: No evidence of invasion of other organs on imaging

T3br: borderline resectable: invasion of other organs (trachea, bronchus, or aorta) cannot be ruled out on imaging. Describe organ(s) suspected of invasion, *e.g.*, cT3br(Trachea)

In this clinical T classification, we abolished the subclassification of cT4. When a tumor is suspected to be resectable without simultaneous resection of adjacent organs, it should be classified as cT3r. When a tumor definitely invades adjacent organs, it is recorded as cT4, even if the adjacent organs are the pleura, pericardium, or diaphragm. When the suspicious target organs of invasion are trachea, bronchus, or aorta, which are usually not resected simultaneously, but no definite findings of cT4 are found, the tumor is classified as cT3br.

*Note 5*: T4 subclassification is used only for pathological diagnosis and not for clinical diagnosis.

pT4a: Pleura, pericardium, diaphragm, lungs, thoracic duct, azygos vein, and nerves

pT4b: Aorta (great artery), trachea, bronchus, pulmonary vein, pulmonary artery, and vertebral body

*Note 6*: Invading organs, such as the pericardium, aorta, vena cava, trachea, lungs, diaphragm, thoracic duct, recurrent laryngeal nerve, and azygos vein, should be recorded, e.g., cT4 (lung), cT4 (aorta), pT4a (lung), pT4b (Trachea).

*Note 7*: When a metastatic lymph node additionally invades a surrounding organ other than the esophagus, it should be classified as T4 and recorded as “T4 (metastatic node number - invaded organ)”, e.g., cT4 (No.112aoA-Aorta).

*Note 8*: Intraductal involvement is defined as pT1a-EP. If the cancer shows invasion outside the duct of the esophageal glands, the depth is defined by the layer of invasion.

*Note 9*: If there are no viable cancer cells capable of proliferating after preoperative treatment, it is classified as T0. In staging, T0 is used equally as T1a.

*e.g.:*CRT-pT0, N0, M0, CRT-pStage 0

*Note 10*: Depth classification of esophageal adenocarcinoma

For cervical (Ce) and thoracic (Te) esophageal adenocarcinoma, follow this Depth of tumor invasion (2.4.1.) ".

Adenocarcinoma of the esophagogastric junction is classified as follows according to the "Gastric Cancer Treatment.”

TX: Depth of tumor unknown

T0: No evidence of primary tumor

T1: Tumor confined to the mucosa (M) or submucosa (SM)

T1a: Tumor confined to the mucosa (M)

   T1a-SMM: Tumor invading the superficial muscularis mucosae (SMM).

   T1a-LPM: Tumor invading the lamina propria mucosa.

   T1a-DMM: Tumor invading the deep muscularis mucosae (DMM). ^*Note 11*^

T1b: Tumor confined to the submucosa (SM)

T2: Tumor invading muscularis propria (MP)

T3: Tumor invading the subserosa (SS)

T4: Tumor invasion is contiguous to or exposed beyond the serosa, or the tumor invading adjacent structures.

   T4a: Tumor invasion is contiguous with the serosa or penetrates the serosa and is exposed in the peritoneal cavity (SE)

   T4b: Tumor invades adjacent structures (AI/SI)

*Note 11*: If a new muscularis mucosae (superficial muscularis mucosae (SMM)) is present, the deep muscularis mucosae (DMM) is regarded as the original muscularis mucosae. When a tumor invades the DMM, it is described as T1a-DMM. If a muscularis mucosal duplication is not found in an esophageal adenocarcinoma, it should be described as T1a-MM.

#### 2.4.2. Diagnostic criteria for depth of superficial esophageal cancer by magnifying endoscopy

The Committee for Diagnostic Criteria of Superficial Esophageal Cancer Depth Using Magnifying Endoscopy of the Japanese Esophageal Association (JESA) has been studying to develop a new, simpler classification based on the two existing endoscopic classifications (Inoue and Arima Classifications). Therefore, we develop a new classification based on narrow-band light-based observation (NBI) and blue laser imaging (BLI), which is simple, objective, and useful for differentiating tumors from non-tumors and evaluating tumor depth.

This classification is based on lesions with territoriality ^*Note 1*^ that are suspected to be squamous cell carcinomas. Vessels seen in borderline lesions ^*Note 2*^ are classified as Type A. Vessels seen in carcinomas are classified as Type B, sub-classified as B1, B2, and B3. The subclassification aims to diagnose the depth of the disease, and the findings in squamous cell carcinomas of T1a-EP and -LPM are classified as Type B1, T1a-MM, and T1b-SM1 as Type B2, and those deeper than T1b-SM2 as Type B3.

**Fig. 9:**
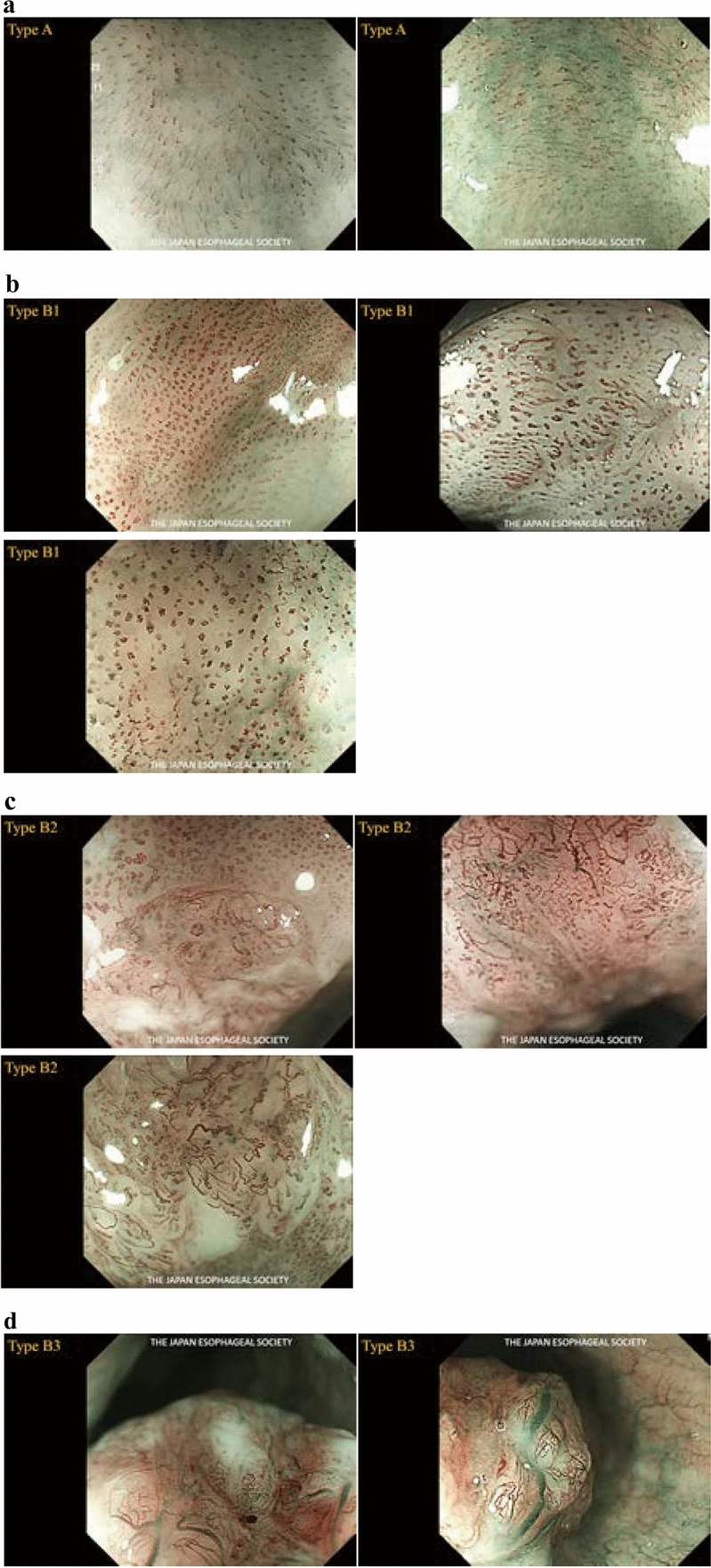

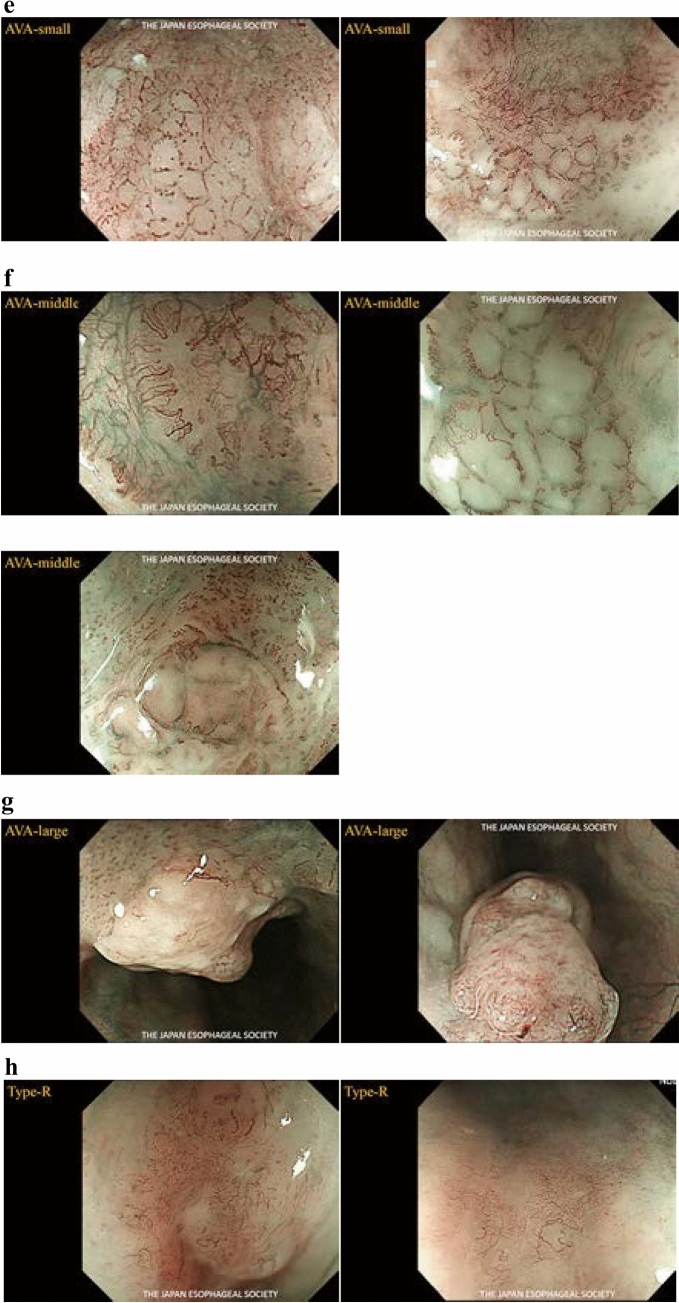
**a** Type A. **b** Type B1. **c** Type B2. **d** Type B3. **e** AVA‒small. **f** AVA‒middle. **g** AVA‒large. **h** Type R

Type A: Normal IPCL ^*Note 3*^ (intraepithelial papillary capillary loop) or abnormal microvessels without severe irregularity (Fig. 9a)

Type B: Abnormal microvessels with severe irregularities or highly dilated abnormal vessels (Fig. 9b–g)

B1: Type B vessels with a loop-like formation (Fig. 9b)^*Note 4*^

B2: Type B vessels without a loop-like formation (Fig. 9c)^*Note 5*^

B3: Highly dilated vessels which calibers (Fig. 9d)^*Note 6*^

Avascular area (AVA): AVA is a certain area without vessels or with sparse vessels surrounded by (any subtypes of) type B microvessels. The AVA is categorized into three types as follows: AVA-small (<0.5 mm in diameter) (Fig. 9e), AVA-middle (0.5 mm or between 0.5 and 3 mm) (Fig. 9f), and AVA-large (≥3 mm) (Fig. 9g). Any type of AVA (small, medium, or large) surrounded by B1 vessels is suggestive of T1a-EP/LPM. An AVA-middle surrounded by B2 or B3 vessels is suggestive of T1a-MM/T1b-SM1. AVA-large surrounded by B2 or B3 vessels is suggestive of T1b-SM2 or deeper.

Reticular pattern vessels are defined as plexiform microvessels and the term "R" is added. This vascular pattern is often found in invasive SCC or non-SCC types of malignant epithelial neoplasms (such as basaloid squamous carcinoma, adenosquamous carcinoma, and neuroendocrine carcinoma) with an infiltrative growth pattern composed of single cells, small tumor nests, or a trabecular arrangement of tumor cells (such as INFc). (Fig. 9h)

The coloration of the epithelium between microvessels presenting as a brownish area (BA: brownish epithelium visualized by NBI or BLI) is defined as intervascular background coloration.

*Note 1*: Lesions whose borders can be followed by normal or image-enhanced observation (dye, digital, and optical digital methods).

*Note 2*: These are mainly squamous intraepithelial neoplasias, but some may include inflammation or carcinoma.

*Note 3*: In normal conditions, the diameter of the vessels is approximately 7–10μm.

*Note 4*: It shows loop-like morphology such as dotted, spiral, or lint-like, with a vessel diameter of approximately 20–30μm.

*Note* 5: Anomalous vessels that do not form loops, such as multilayered (ML) and irregularly branched (IB) vessels.

*Note 6*: Irregular vessels that are approximately 3 times larger than B2 vessels and have a vessel diameter greater than approximately 60µm.

#### 2.4.3 Diagnosis of cT4 invasion of adjacent organs by CT and other imaging techniques (Figs. 10, 11, 12, 13, 14, 15, 16, 17, 18, 19, 20, 21)

**Fig. 10:**
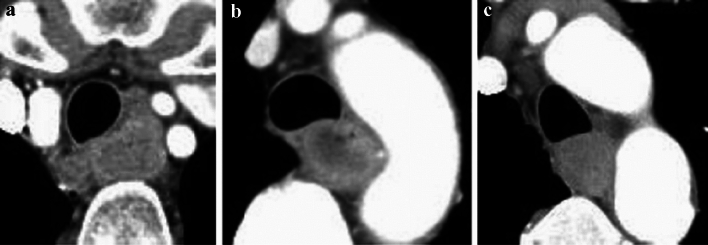
Cases of the primary lesion contacting the trachea. **a** Type 2, circumference, and posterior wall. **b** Type 2, circumference, and posterior wall. **c** Type 2, 2/3 of the circumference, and left-anterior wall

**Fig. 11:**
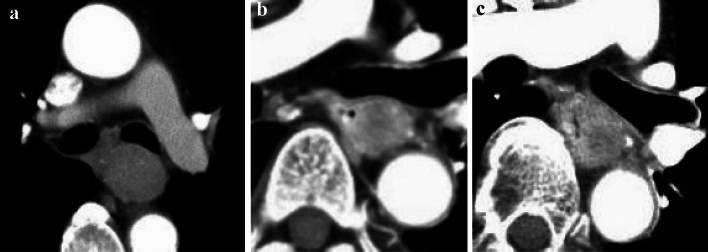
Cases of the primary lesion contacting the left main bronchus. **a** Type 2, circumference, and posterior wall. **b** Type 1, circumference, and left wall. **c** Type 2, circumference, and posterior wall

**Fig. 12:**
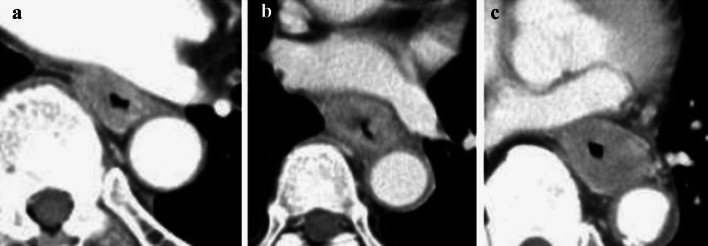
Cases contacting the aorta. **a** Type 3, 1/3 of the circumference, and right wall. **b** Type 2, 2/3 of the circumference, and right wall. **c** Type 2, sub-circumference, and right wall

**Fig. 13:**
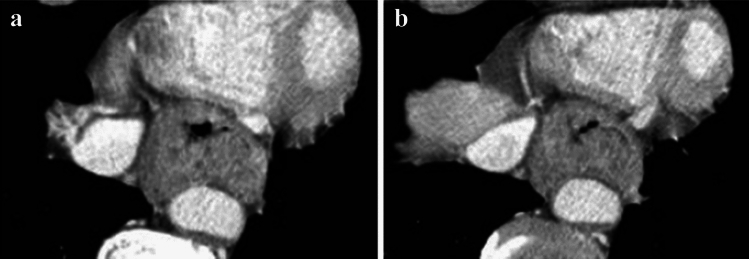
**a** and **b** A case whose invasion to the aorta is not ruled out (**a** and **b** are of the same case)

**Fig. 14:**
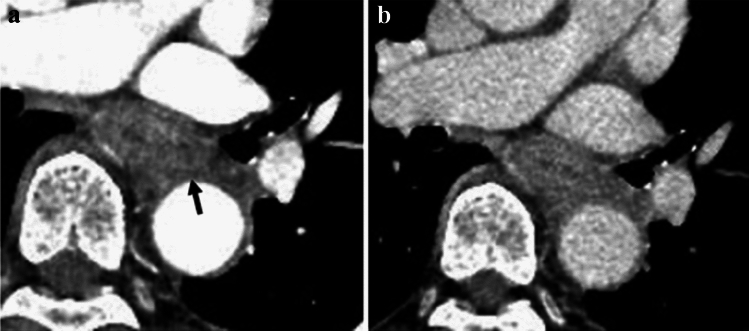
**a** and **b** A case whose invasion to the aorta is not ruled out (**a** and **b** are of the same case)

**Fig. 15:**
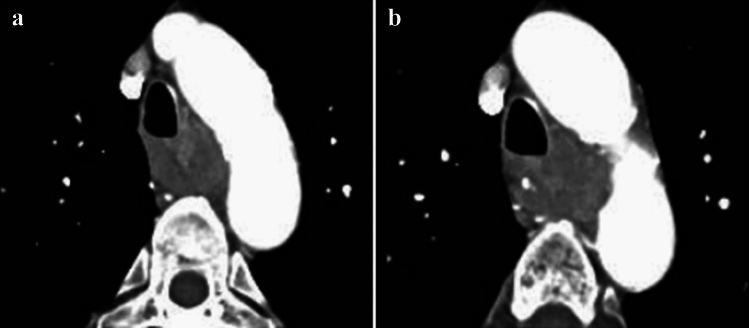
**a** and **b** A case whose invasion to the trachea is not ruled out (**a** and **b** are of the same case)

**Fig. 16:**
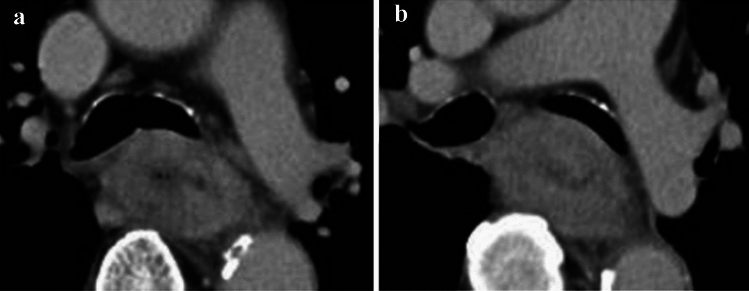
**a** and **b** A case with suspicion of invasion to the left main bronchus (**a** and **b** are of the same case)

**Fig. 17:**
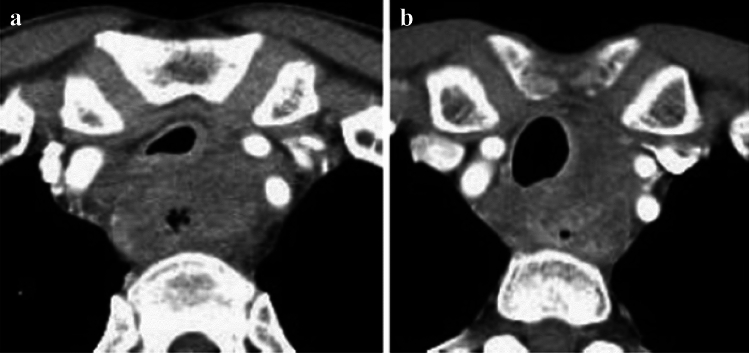
**a** and **b** Cases with invasions to the trachea

**Fig. 18:**
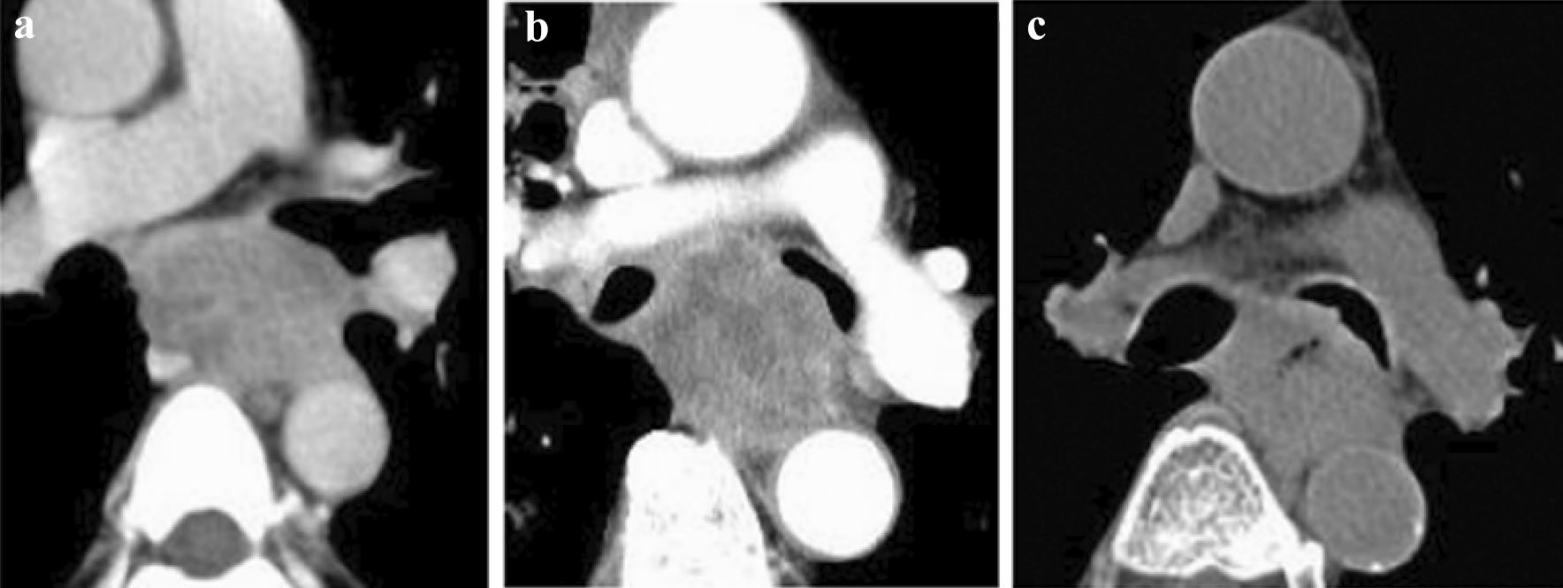
**a**–**c** Cases with invasions to the left main bronchus

**Fig. 19:**
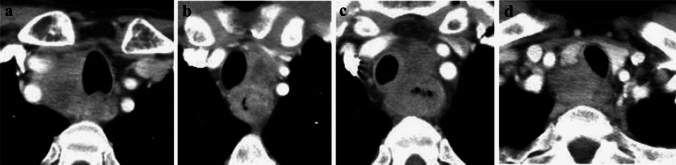
**a**–**d** Cases with invasions to the trachea from metastatic lymph nodes

**Fig. 20:**
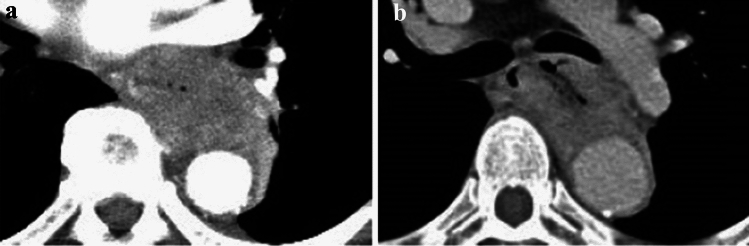
**a** and **b** Cases with invasions to the aorta

**Fig. 21:**
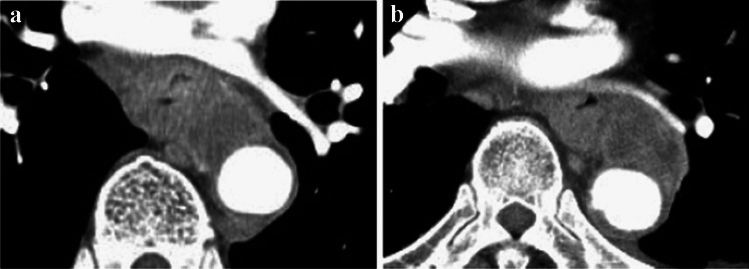
**a** and **b** Cases with invasions to the aorta from metastatic lymph nodes

This classification system has abolished subclassification of T4 in the former edition as a clinical diagnosis. However, in practice, it remains unclear whether the tumor is cT3 or cT4, or resectable or unresectable. In cases of obvious adjacent organ involvement, such as an esophagotracheal fistula, the diagnosis of cT4 is easy; however, the involvement of adjacent organ is often difficult to diagnose. Clinically, the question is whether R0 can be achieved without resection of the adjacent organs (trachea, bronchus, and aorta). Therefore, we subclassified cT3 into two, cT3br (borderline resectable), wherein the invasion of adjacent organs cannot be ruled out, and cT3r (resectable), wherein the invasion of adjacent organs can be ruled out. When a tumor is considered to invade the adjacent organs, it is recorded as cT4 even if the adjacent organs are the pleura, pericardium, or diaphragm. When a tumor might invade the adjacent organs (such as the trachea, bronchus, or aorta) wherein the organs are not usually resectable but no definite findings of cT4 are found, the tumor is classified as cT3br.

Tracheal and bronchial invasion is diagnosed when the stratified structure of the border of contact with the airway by the primary tumor or lymph node metastases is unclear and when the airway is compressed, displaced, or the lumen is narrowed. In addition to CT images, it is also important to evaluate bronchoscopy findings.

Aortic invasion is diagnosed when the layered structure of the contact border with the aorta by the primary tumor or lymph node metastases is unclear, and the contact angle (Picus angle) at ≥ 110 degrees can be detected with ≥ 10 mm in cephalocaudal length, or the angle of contact (Picus angle) at 90–110 degrees can be detected with ≥ 20 mm in cephalocaudal length. In addition, if the tumor contour collapses and contacts or encircles the aorta, aortic invasion is diagnosed.

In addition to enhanced CT, upper gastrointestinal endoscopy and esophagography are used to make a cT4 diagnosis, considering the primary tumor's main wall location (such as posterior wall predominant or sub-total) and the gross type of the primary tumor (bulge or ulcer). If necessary, MRI and EUS are used to aid in the diagnosis of the aortic and tracheal wall structures.

In this chapter, typical CT images of cT3r, cT3br, and cT4 are presented for reference. Regarding each CT image, the rates of agreement of diagnosis among the experts are shown.

##### 2.4.3.1. Typical CT images: cT3r (Figs. 10, 11, 12)

We present typical CT images of cT3r that do not invade the adjacent organs. We provide the rates of agreement of the tumor depth by seven experts who belong to the working group of the Committee of Japanese Classification of Esophageal Cancer as reference data.

The laterality of the primary lesion on the circumference is significant in the diagnosis of tracheal invasion. Although the tracheal membrane is often compressed by tumors of the upper esophagus, the possibility of tracheal invasion differs between anterior and posterior tumors. In these three figures, the primary lesions (located in the upper esophagus) appear to contact the tracheal membrane or tracheal cartilage. As shown in Fig. 10a and b, the tracheal invasion is not determined because the tumors are located in the posterior wall and low-density layers between the trachea, and an enhanced outline of the tumors can be detected. As shown in Fig. 10c, we determine no tracheal invasion because the boundary of the trachea is clear and no compression, dislocation, or stenosis is observed despite the presence of a left-anterior tumor.

Agreement of diagnosis of cT3: 10a, 85.7%, 10b, 100%, 10c, 85.7%

The laterality of the primary lesion in the circumference is also significant for the diagnosis of invasion of the left main bronchus. In these three figures, the primary lesions (located in the middle esophagus) contact the left main bronchial membranes and appear to compress them slightly. However, all three patients are diagnosed as having no main bronchial invasion because low-density layers between the left main bronchus and the enhanced outline of the tumors can be detected, there are no findings of bronchial stenosis or deformity, and the tumors are located in the posterior wall.

Agreement of diagnosis of cT3: 11a, 100%, 11b, 100%, and 11c, 100%.

For the diagnosis of aortic invasion, the laterality of the primary lesion in the circumference is also significant. In these three figures, the primary lesions (located in the middle or lower esophagus) are in contact with the descending aorta. All three patients are diagnosed with no aortic invasion because the fat layers between the primary lesions and the aorta can be clearly detected, and their angles of contact (Picus’ angle) are < 90°[7].

Agreement of diagnosis of cT3: 12a, 100%, 12b, 71.4%, 12c, 100%

##### 2.4.3.2 Typical CT images: cT3br (Figs. 13, 14, 15, 16)

We present typical CT images of cT3br, which are not ruled out to invade the adjacent organs.

In this case, the angle of contact (Picus’ angle) ≥ 110 degrees can be detected with ≥ 10 mm in cephalocaudal length and compression to the aorta is also found. However, the outline of the tumor contacting the aorta remains, and a low-density layer between the aorta and the enhanced outline of the tumor can also be detected. Overall, with a suspicion of cT4, this case is diagnosed as cT3br [7].

Agreement of diagnosis of cT3: 57.1%

In this case, the angle of contact (Picus’ angle) ≥ 110 degrees can be detected with ≥ 10 mm in cephalocaudal length and the tumor surrounds the aorta. However, infiltrative shadows are believed to be caused by edema or other effects because most of the esophageal wall is confirmed to be in the arterial phase. Finally, with the suspicion of cT4, this patient is diagnosed as cT3br [7].

Agreement of diagnosis of cT3: 57.1%

In this case, the primary lesion (located in the upper esophagus) was found to be in contact with a large area of the tracheal membrane and cartilage, as well as the surrounding areas; however, there are no findings of tracheal compression, dislocation, stenosis, or deformation. Therefore, with the suspicion of cT4, this case is diagnosed as cT3br.

Agreement of diagnosis of cT3: 57.1%

Although the primary lesion in the upper esophagus contacts the left main bronchus, compression, dislocation, stenosis, and deformation of the left main bronchus are relatively mild and smooth. Therefore, with the suspicion of cT4, this case is finally diagnosed as cT3br.

Agreement of diagnosis of cT3: 57.1%

##### 2.4.3.3. Typical CT images: cT4 (Figs. 17, 18, 19, 20, 21)

We present typical CT images of cT4, which are considered to definitely invade adjacent organs.

The primary lesions (located in the upper esophagus) are in contact with the tracheal membrane and part of the tracheal cartilage. Based on the findings of tracheal compression, dislocation, stenosis, and deformation, these patients are diagnosed with invasions to the trachea.

Diagnostic agreement of cT4:17a, 85.7%, 17b, 85.7%

The primary lesions (located in the middle esophagus) contact the left main bronchus, and compression of the bronchial membrane, bronchial stenosis, and deformation are observed. These patients are diagnosed with invasions to the left main bronchus.

Agreement of diagnosis of cT4: 18a, 85.7%, 18b, 85.7%, 18c, 85.7%.

Metastatic nodes of 106recR or 106recL contact the right and left tracheal cartilage and the tracheal membrane widely. Tracheal compression and dislocation are observed. These cases are diagnosed with invasion to the trachea (cT4).

Agreement of diagnosis of cT4: 19a, 100%, 19b, 100%, 19c, 85.7%, 19d, 100%

The boundary layers between the primary lesions (located in the lower esophagus) and the aorta are obscure, and the angles of contact (Picus’ angle) are 160° (Fig. 16a) and 110° (Fig. 16b). These lesions appear to surround the aorta, and these findings are confirmed in cranial and caudal images (data not shown). These patients are diagnosed with cT4 [7].

Agreement of diagnosis of cT4: 20a, 85.7%, 20b, 71.4%

The metastatic lymph nodes of 108 located between the primary lesions (middle esophagus) and the aorta contact with the aorta, losing the boundary of the nodes. Boundary layers could not be detected. The angles of contact (Picus’ angle) are 100° (Fig. 21a) and 120° (Fig. 21b) and these findings are confirmed in the cranial and caudal images (Figures not shown). These cases are diagnosed with invasion to the aorta [7].

Agreement of diagnosis of cT4: 21a, 85.7%, 21b, 71.4%

### 2.5. Infiltrative growth pattern (INF)

Infiltrative growth patterns of tumors can be classified into one of the following three types based on the predominant pattern observed at the tumor margins.

INFa (expansive type): expansive growth of tumor nests with a well-demarcated border from the surrounding tissue.

INFb (intermediate type): intermediate growth pattern, between INFa and INFc.

INFc (infiltrative type): infiltrative growth of tumor nests with an ill-defined border from the surrounding tissue.

### 2.6. Lymphatic or venous invasion

These descriptions (Ly or V) are used only for pathological findings and not for clinical findings.

*Note 1*: Indefinite discrimination of lymphatic or venous invasion is described as Ly/V.

#### 2.6.1 Lymphatic invasion (Ly)

- LyX: lymphatic invasion cannot be assessed.
- Ly0: None.
- Ly1: Lymphatic invasion is observed.
  - Ly1a: Slight, one or two lymphatic vessels are involved.
    Ly1b: Moderate.
    Ly1c: Severe.

*Note:* A tumor mass found in the thoracic duct is described as positive for lymphatic invasion.

#### 2.6.2 Venous invasion (V)

- VX: Venous invasion cannot be assessed.
- V0: None.
- V1: Venous invasion is observed.
  - V1a: Slight, one or two veins are involved.
    V1b: Moderate.
    V1c: Severe.

*Note:* For endoscopically resected specimens, it is not necessary to evaluate the degree of vascular involvement; only its presence or absence should be described.

Lymphatic invasion (Ly)

- LyX: lymphatic invasion could not be assessed
- Ly0: None
- Ly1: Lymphatic invasion is observed

Venous invasion (V)

- VX: Venous invasion could not be assessed
- V0: None
- V1: Venous invasion is observed

### 2.7. Description of surgical findings

#### 2.7.1. Tumor size (Fig. 22)

**Fig. 22:**
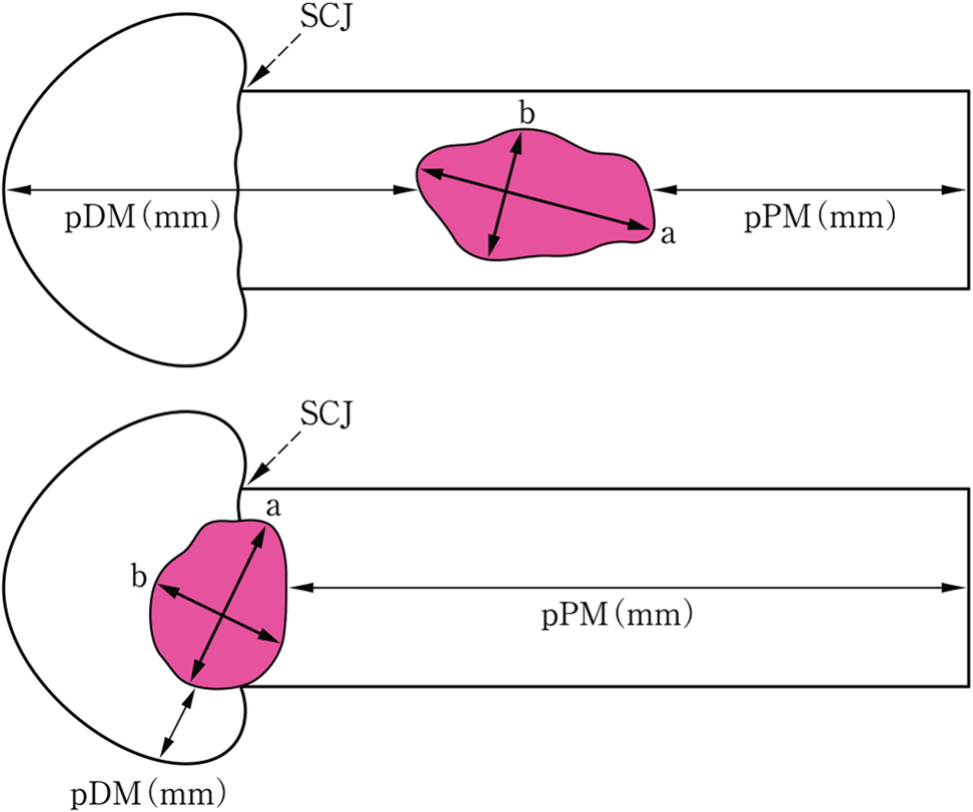
Tumor size and the distance from resection margin to tumor. **a** Greatest longitudinal dimension (mm). **b** Greatest transverse dimension (mm)

Greatest longitudinal dimension (mm)

Greatest transverse (at 90° to the longitudinal tumor axis) dimension (mm)

#### 2.7.2. Distance from surgical margin to the tumor (Fig. 22)

Proximal (oral) margin (PM) (mm)

Distal (anal) margin (DM) (mm)

#### 2.7.3. Surgical tumor type (refer to 2.3.)^*Note*^

The macroscopic appearance of tumors before and after fixation may differ. Under such circumstances, the surgical tumor type should be described according to pre-fixation observations, and the pathological tumor type should be described based on post-fixation findings. Pathological tumor types can be classified based on cross-sectional observations. Surgical tumor types should be determined regardless of the pathological depth of tumor invasion.

*Note*: The presence of preoperative chemotherapy and radiotherapy should be recorded for surgical tumor types.

#### 2.7.4 Surgical margin

Cancer invasion at the resection margin is evaluated pathologically, and a p- is assigned to each finding. The pathological and surgical findings are judged comprehensively as the final findings (f).

##### 2.7.4.1 Proximal margin (PM)^*Note*^

- PMX: Proximal margin cannot be assessed
- PM0: No evidence of tumor invasion
- PM1: Tumor invasion present at the proximal margin

*Note*: The distance from the resection margin to the tumor is recorded in millimeters for the PM0 specimens.

##### 2.7.4.2 Distal margin (DM)^*Note*^

- DMX: Distal Margin cannot be assessed
- DM0: No evidence of tumor invasion
- DM1: Tumor invasion present at the distal margin

*Note*: The distance from the resection margin to the tumor is recorded in millimeters for DM0 specimens.

##### 2.7.4.3 Radial margin (RM)^*Note*^

- RMX: Radial margin cannot be assessed
- RM0: No evidence of tumor invasion
- RM1: Tumor invasion present at the radial margin

*Note*: The distance from the resection margin to the tumor is recorded in millimeters for RM0 specimens.

### 2.8. Description of endoscopic findings

#### 2.8.1. Number of tumors and resected specimens

Number of lesions

Number of specimens resected from each lesion:

e.g., 1. en bloc resection; 2. Divided resection (divided resection means that the lesion was resected but not in en bloc fashion. In this case, the number of divided specimens should be recorded.)

#### 2.8.2. Size of resected specimen and size of each tumor lesion

Size is described as the greatest longitudinal dimension in millimeters multiplied by the greatest transverse dimension in millimeters: a × b (mm).

#### 2.8.3. Tumor types

The tumor types are classified as Type 0-Ip, Type 0-Is, Type 0-IIa, Type 0-IIb, Type 0-IIc, Type 0-III, combined type, and others.

#### 2.8.4 Resection margin^*Note1, 2*^

Cancer invasion at the resection margin is evaluated pathologically, and a p- is assigned to each finding. The pathological and endoscopic findings are judged comprehensively as the final findings (f).

##### 2.8.4.1 Horizontal margin (HM)^*Note 3*^

- HMX: The presence or absence of residual tumors in the horizontal margin cannot be assessed.
- HM0: Non-cancerous squamous epithelium and lamina propria mucosae confirmed on all horizontal resection margins.
- HM1: The tumor is exposed on any horizontal resection margin.

##### 2.8.4.2 Vertical margin (VM)

- VMX: Residual tumor in the vertical margin cannot be assessed.
- VM0: No tumor is exposed on any vertical margin.
- VM1: The tumor is exposed on any vertical margin.

*Note 1*: When no tumor is recognized in any resection margin, it is defined as complete resection (pR0); when a tumor is recognized in any resection margin, it is defined as incomplete resection (pR1).

*Note 2*: The presence of vascular invasion in the resection margin is defined as a positive resection margin (pHM1 or pVM1).

*Note3*: When a lesion is resected in divided fashion, outer margin of the reassembled specimens should be regarded as horizontal margin.

### 2.9. Multiple primary cancers

#### 2.9.1. Multiple primary cancers of the esophagus

The term “multiple primary cancers of the esophagus” refers to the presence of two or more primary esophageal cancers that are located separately from each other. They should be described separately from intramural metastatic lesions. If multiple primary cancers are present, the location, size, macroscopic findings, and tumor depth should be described for each lesion. Among multiple lesions, the primary lesion is assigned to the lesion with the deepest depth or the lesion with the largest diameter if the depths are the same.

*Note 1*: Descriptions of the locations of multiple primary cancers of the esophagus should be made according to the order of the depth of tumor invasion (deeper to shallower), inserting “/” between the abbreviations for the location of each lesion; the total number of lesions should also be recorded in parentheses. For example, MtUt/Lt/Lt (3 lesions)

*Note 2*: Secondary lesions that are clearly histologically different from the main lesion or those with the same histology but with evidence of intraepithelial carcinoma are considered primary lesions and regarded as multiple carcinomas.

#### 2.9.2. Multi-organ primary cancers including the esophagus

The term “multi-organ primary cancers including the esophagus” refers to the presence of one or more primary malignant diseases other than esophageal cancer in patients with primary esophageal cancer.

*Note 1*: In cases of multi-organ primary cancers including the esophagus, organs other than the esophagus should be specified in parentheses.

*Note 2*: Whether the multiplicity is synchronous or metachronous should be recorded, e.g., multi-organ primary cancers: stomach (synchronous).

Cancers diagnosed within a period of <1 year are considered synchronous cancers.

Cancers diagnosed over a period of ≥1 year are considered metachronous cancers.

If both simultaneous and metachronous cancers are present, they are considered synchronous and metachronous.

#### 2.10. Intramural metastasis (IM)

Metastatic lesions in the esophagus, pharynx, or gastric wall macroscopically (clearly) separated from the primary tumor should be recorded as IM, and the number of such lesions should be described.

- IMX: Intramural metastasis cannot be assessed
- IM0: No intramural metastasis.
- IM1: Intramural metastasis.

*Note*: IM in the gastric wall should be recorded as “IM1-St.” This is classified as organ metastasis (M1).

## 3. Description of lymph nodes

### 3.1. Name, number, and extent and boundaries of lymph node station in esophageal cancer

**Fig. 23:**
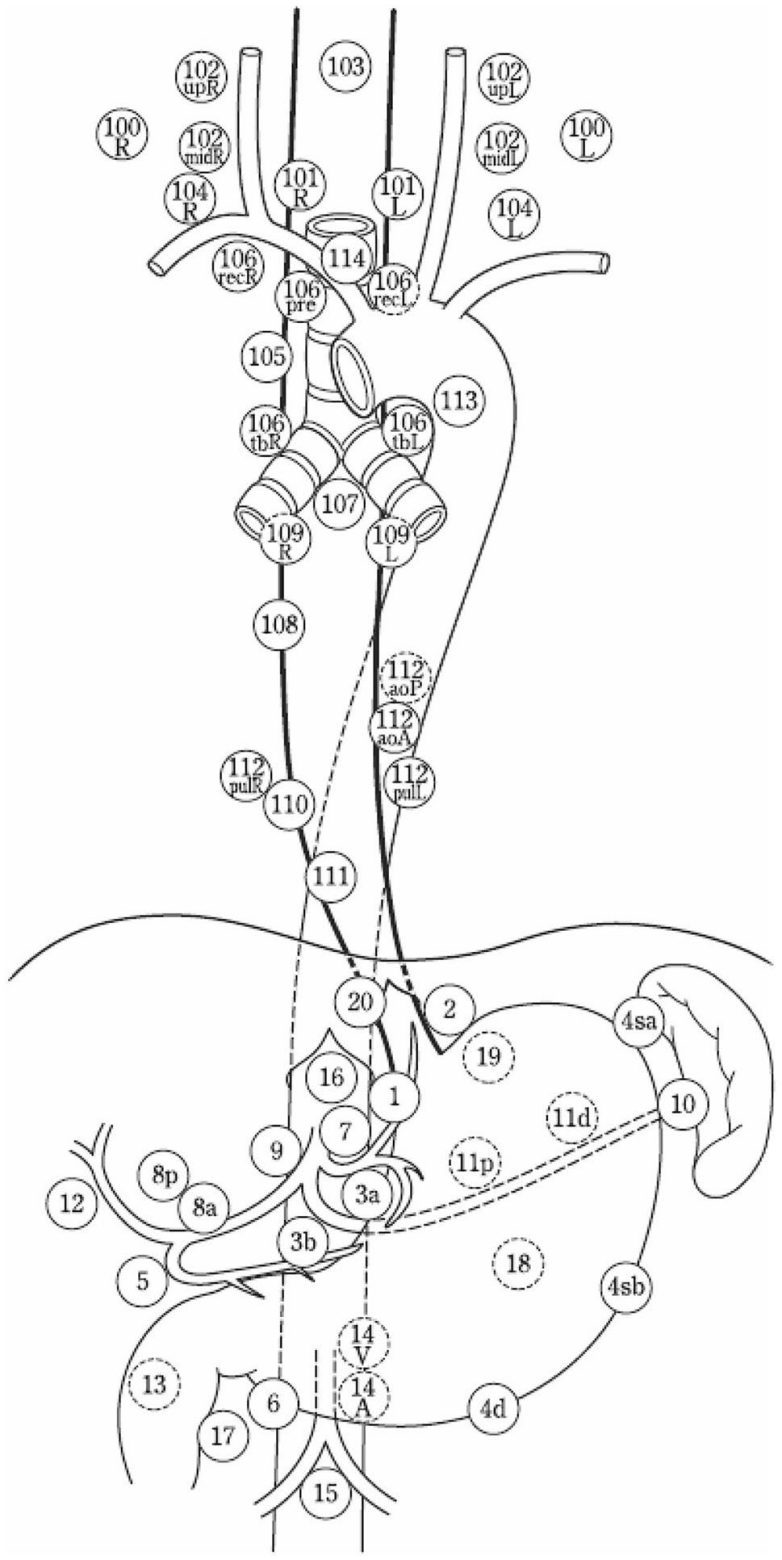
Station numbers of regional lymph nodes

The names and numbers of lymph nodes are defined in Table 5 and Fig. 23 (refer to 19 in Part II).

**Table 5:** Numbers and naming of regional lymph nodes

(1) Cervical lymph nodes	
No.100	Superficial lymph nodes of the neck
No.100spf	Superficial cervical lymph nodes
No.100sm	Submandibular lymph nodes
No.100tr	Cervical pretracheal lymph nodes
No.100ac	Accessory nerve lymph nodes
No.101	Cervical paraesophageal lymph nodes
No.102	Deep cervical lymph nodes
No.102up	Upper deep cervical lymph nodes
No.102mid	Middle deep cervical lymph nodes
No.103	Peripharyngeal lymph nodes
No.104	Supraclavicular lymph nodes
(2) Thoracic lymph nodes	
No.105	Upper thoracic paraesophageal lymph nodes
No.106	Thoracic paratracheal lymph nodes
No.106rec	Recurrent nerve lymph nodes
No.106recL	Left recurrent nerve lymph nodes
No.106recR	Right recurrent nerve lymph nodes
No.106pre	Pretracheal lymph nodes
No.106tb	Tracheobronchial lymph nodes
No.106tbL	Left tracheobronchial lymph nodes
No.106tbR	Right tracheobronchial lymph nodes
No.107	Subcarinal lymph nodes
No.108	Middle thoracic paraesophageal lymph nodes
No.109	Main bronchus lymph nodes
No.109L	Left main bronchus lymph nodes
No.109R	Right main bronchus lymph nodes
No.110	Lower thoracic paraesophageal lymph nodes
No.111	Supradiaphragmatic lymph nodes
No.112	Posterior mediastinal lymph nodes
No.112aoA	Anterior thoracic paraaortic lymph nodes
No.112aoP	Posterior thoracic paraaortic lymph nodes
No.112pul	Pulmonary ligament lymph nodes
No.113	Ligamentum arteriosum lymph nodes (Botallo lymph nodes)
No.114	Anterior mediastinal lymph nodes
(3) Abdominal lymph nodes	
No.1	Right paracardial lymph nodes
No.2	Left paracardial lymph nodes
No.3a	Lesser curvature Lymph nodes along the branches of the left gastric artery
No.3b	Lesser curvature Lymph nodes along the 2nd branches and distal part of the right gastric artery
No.4	Lymph nodes along the greater curvature
No.4sa	Lymph nodes along the short gastric vessels
No.4sb	Lymph nodes along the left gastroepiploic artery
No.4d	Lymph nodes along the right gastroepiploic artery
No.5	Suprapyloric lymph nodes
No.6	Infrapyloric lymph nodes
No.7	Lymph nodes along the left gastric artery
No.8a	Lymph nodes along the common hepatic artery (Anterosuperior group)
No.8p	Lymph nodes along the common hepatic artery (Posterior group)
No.9	Lymph nodes along the celiac artery
No.10	Lymph nodes at the splenic hilum
No.11	Lymph nodes along the splenic artery
No.11p	Lymph nodes along the proximal splenic artery
No.11d	Lymph nodes along the distal splenic artery
No.12	Lymph nodes in the hepatoduodenal ligament
No.13	Lymph nodes on the posterior surface of the pancreatic head
No.14	Lymph nodes along the superior mesenteric vessels
No.14A	Lymph nodes along the superior mesenteric artery
No.14V	Lymph nodes along the superior mesenteric vein
No.15	Lymph nodes along the middle colic artery
No.16	Lymph nodes around the abdominal aorta
No.16a1	Lymph nodes in the aortic hiatus
No.16a2	Lymph nodes around the abdominal aorta (from the upper margin of the celiac trunk to the lower margin of the left renal vein)
No.16b1	Lymph nodes around the abdominal aorta (from the lower margin of the left renal vein to the upper margin of the inferior mesenteric artery)
No.16b2	Lymph nodes around the abdominal aorta (from the upper margin of the inferior mesenteric artery to the aortic bifurcation)
No.17	Lymph nodes on the anterior surface of the pancreatic head
No.18	Lymph nodes along the inferior margin of the pancreas
No.19	Infradiaphragmatic lymph nodes
No.20	Lymph nodes in the esophageal hiatus of the diaphragm

*Note:* The left side (L) and the right side (R) should be distinguished for 101, 102, 104, 106rec, 106tb, 109, and 112pul

The names and numbers of the abdominal lymph node stations are defined according to the Japanese Classification of Gastric Carcinoma [8].

*Note 1*: The number of lymph node stations should be recorded using “No.” plus a number, e.g., No.106recR.

*Note 2*: Imaging criteria for the diagnosis of lymph node metastasis using CT and PET/CT (refer to 20 in Part II)

- For the diagnosis of lymph node metastasis by CT, the slice size should be ≤ 2.5 mm on contrast-enhanced CT.
- Based on the analysis limited to lymph nodes of short diameter ≥ 5 mm of patients with cT2-T4, a short diameter of 6 mm on CT is recommended as the cut-off criterion. However, there are limitations to diagnosing lymph node metastases based on size alone, since approximately 1/3 of metastatic lymph node have a short diameter of < 5 mm.
- Although PET/CT is superior to CT in terms of the positive predictive value of lymph node metastasis, the diagnostic criteria for positive SUV have not yet been standardized due to inter-institutional differences and the likelihood of being affected by many factors (patient’s blood sugar level, type of machine, or protocol).
- On PET-CT, it should be noted that lymph nodes in the mediastinum, especially those in the hilar region, can accumulate FDG due to inflammation in the lung field or sarcoidosis, resulting in false-positive results.

*Note 3:* The numbers of metastatic and resected nodes are recorded at each lymph node station, e.g., No.104R (0/10), No.104L (1/13), No.106recL (0/4).

*Note 4:* Extralymph node metastasis (tumor nodule), a cancer nest without lymphatic tissue in the fat tissue outside the esophagus or stomach, is confirmed, and its number and station should be recorded as extralymph node metastasis.

*Note 5:* Extranodal involvement (direct or vascular invasion) should be recorded.

### 3.2 Regional lymph nodes

**Fig. 24:**
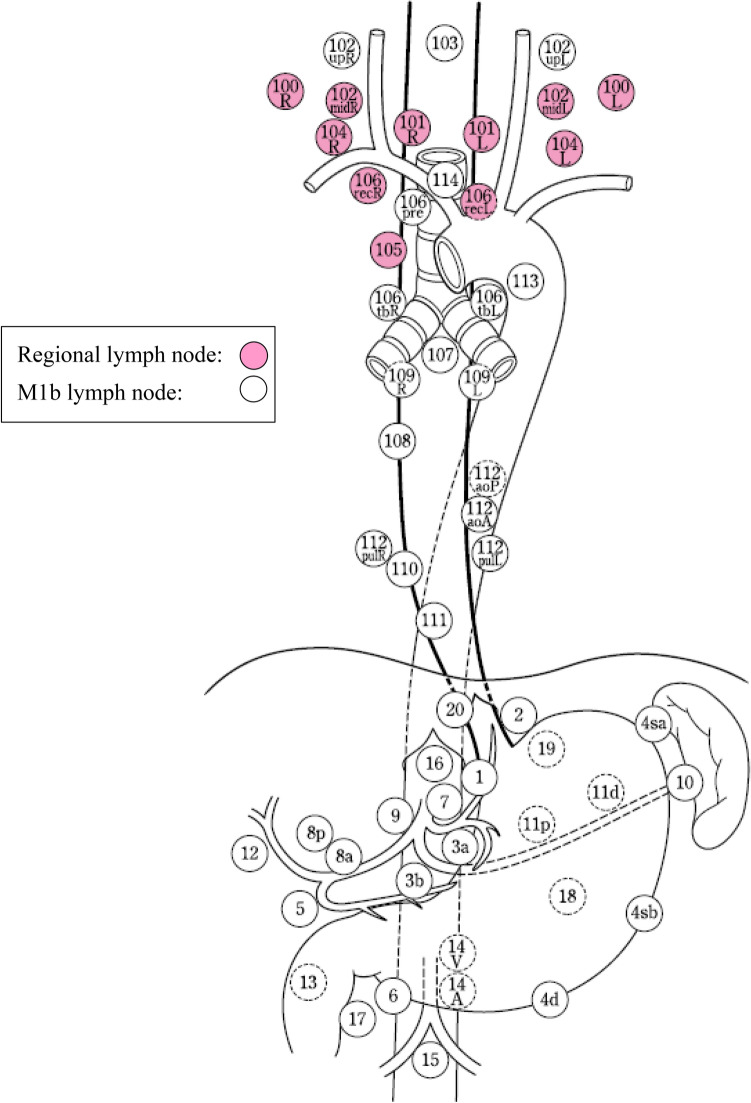
Regional lymph nodes of cervical esophageal cancer (Ce)

**Fig. 25:**
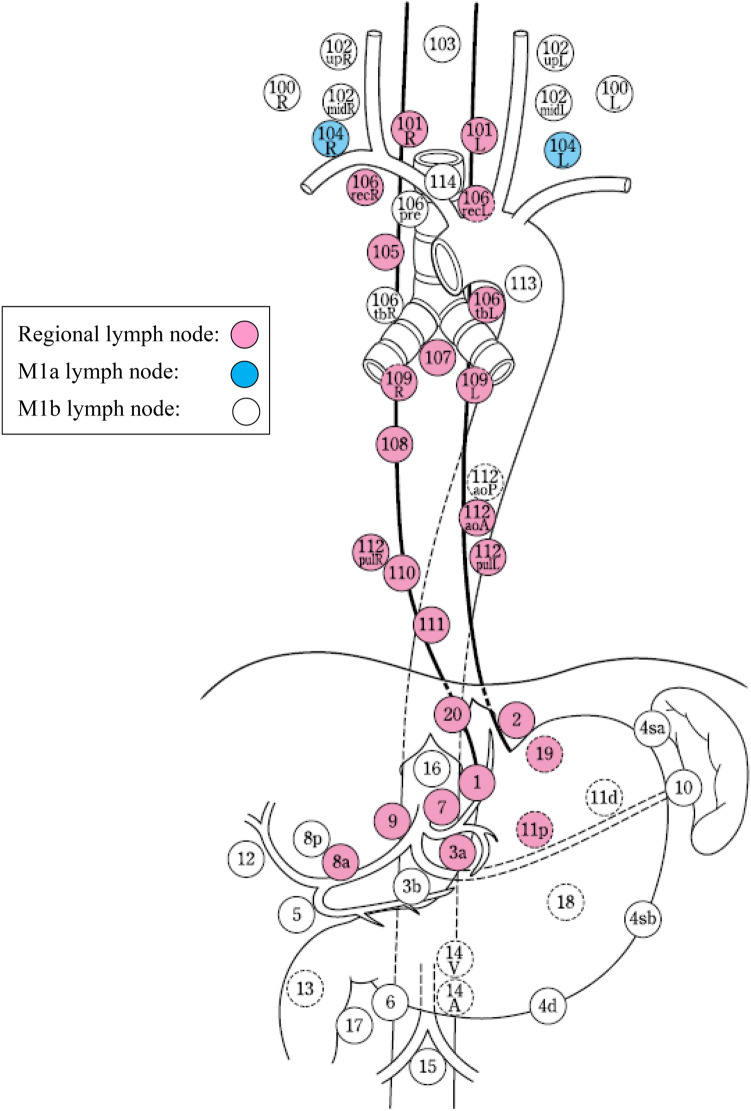
Regional lymph nodes of thoracic esophageal cancer (Te)

**Fig. 26:**
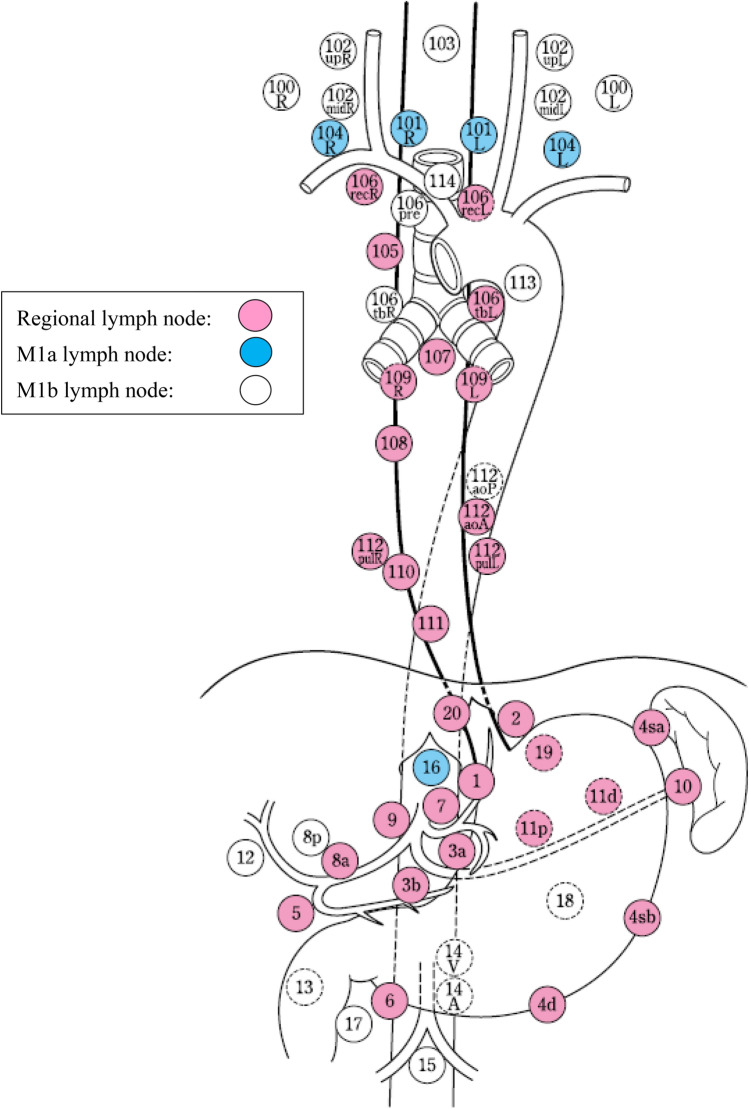
Regional lymph nodes of zone of esophagogastric junction cancer (Jz)

Regional lymph nodes are defined according to the location of the tumor (Ce, Te, and Jz), as shown in Table 6 and Figs. 24, 25, 26.

*Note 1*: For multiple esophageal cancers or tumors extending continuously into more than one portion of the esophagus, the regional lymph nodes should be determined based on the location of the deepest tumor invasion or the main tumor location.

*Note 2*: Until the 11th edition of the "Japanese Classification of Esophageal Cancer,” the degree of lymph node metastasis was classified according to the site of metastatic lymph nodes. However, in this edition, the N classification is changed to a system based on the number of metastatic lymph nodes in accordance with the TNM classification of the Union for International Cancer Control (UICC). We decide on the extent of the regional lymph nodes so that patients can be expected to have a survival benefit from the dissection of these nodes. The degree of lymph node metastasis (N1-N3) is determined based on the number of metastatic nodes in the regional lymph nodes.

*Note 3*: The concept of regional lymph nodes

**Table 6:** Lymph node groups according to the tumor location

Tumor location	Regional lymph nodes	M1a	M1b
Cervical (Ce)	Cervical: 100, 101, 102mid, 104 Thoracic: 105^*Note*^, 106rec^*Note*^	None	Other lymph nodes
Thoracic (Te)	Cervical: 101 Thoracic: 105, 106rec, 106tbL, 107, 108, 109, 110, 111, 112aoA, 112pul Abdominal: 1, 2, 3a, 7, 8a, 9, 11p, 19, 20	104	102 106pre, 106tbR, 112aoP, 16, other lymph nodes
Zone of esophagogastric junction (Jz)	Cervical: none Thoracic: 105, 106rec, 106tbL, 107, 108, 109, 110, 111, 112aoA, 112pul Abdominal: 1, 2, 3a, 3b, 4sa, 4sb, 4d, 5, 6, 7, 8a, 9, 10, 11p, 11d, 19, 20	101, 104, 16	Other lymph nodes

*Note*: Limited to the area which can be dissected from the cervical incision

- The supraclavicular lymph nodes (No. 104) in thoracic esophageal cancer and No. 101, 104, and 16 in the esophagogastric junction (Jz) cancer are extra-regional lymph nodes; however, we recognize them as M1a to distinguish them from other M1b nodes because some dissection benefit can be expected.
- The M1a lymph nodes (No.104 for thoracic esophageal cancer and No.101, 104, and 16 for esophagogastric junction cancer) described above and other extra-regional lymph nodes (M1b) are not included in the count of the number of regional lymph node metastases.
- Lymph nodes 102, 106pre, 106tbR, and 112aoP in thoracic esophageal cancer are classified as extra-regional lymph nodes (M1b) because the effect of prophylactic dissection is unknown, although they are occasionally dissected in metastatic cases.

### 3.3. Grading of lymph node metastasis (N)

- NX: Regional lymph node metastasis cannot be assessed
- N0: No regional lymph node metastasis
- N1: Metastasis to 1 to 2 regional lymph nodes
- N2: Metastasis to 3 to 6 regional lymph nodes
- N3: Metastasis to 7 or more regional lymph nodes

For pathological diagnosis of lymph node metastasis, D2 or more lymphadenectomy for cervical and thoracic esophageal cancer and D1 + α or more lymphadenectomy for esophagogastric junction cancer is preferable (refer to 5.3.3).

## 4. Distant organ and lymph node metastasis (M)

- MX: Distant organ or extra-regional lymph node metastasis cannot be assessed
- M0: Neither distant organ nor extra-regional lymph node metastasis
- M1a: Metastasis in lymph node that is outside the region but can be expected to have some efficacy in dissection
- M1b: Metastasis to lymph node that are neither regional lymph node nor M1a, or metastasis to distant organ

*Note:* Refer to 3.2 Regional lymph nodes

Distant organ metastasis should be determined through comprehensive consideration of operative macroscopic findings, intraoperative imaging examinations such as intraoperative ultrasound examination, intraoperative pathological diagnosis with frozen sections, and final pathological findings. Whether distant organ metastases are resected should be recorded.

*Note 1:* The site of distant metastasis should be recorded as follows: lymph node (LYM), skin (SKI), liver (HEP), lung (PUL), bone marrow (MAR), bone (OSS), peritoneum (PER), pleura (PLE), brain (BRA), meninx (MEN), adrenal (ADR), and others (OTH).

*Note 2:* Intramural metastasis to the stomach is regarded as distant metastasis and recorded as M1b (IM1-St).

## 5. Stage (Tables 7, 8, 9, 10)

T, N, and M category and stage is recorded, e.g., T2N2M0, Stage IIIA.

The stages are divided into two categories: clinical stage based on diagnostic imaging and pathological stage based on histological examination of the resected specimen.

### 5.1 Staging of cervical and thoracic esophageal cancer, and squamous cell carcinoma of the esophagogastric junction

**Table 7:**
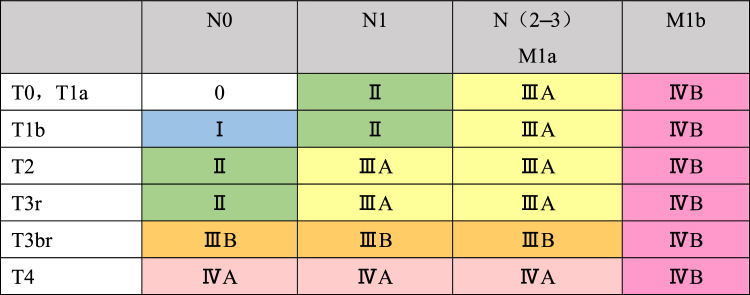
Clinical stage

**Table 8:**
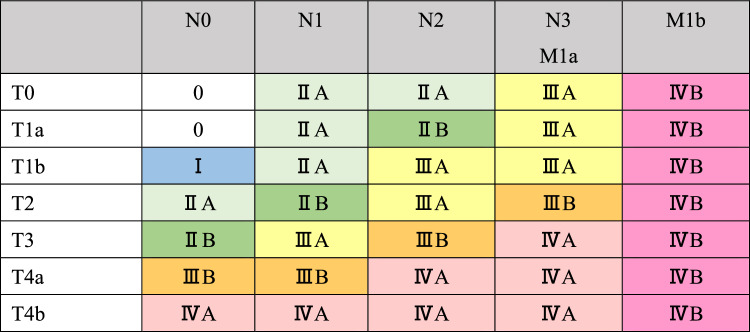
Pathological stage

### 5.2 Staging of adenocarcinoma of the esophagogastric junction

The staging of esophagogastric junction adenocarcinoma should conform to that of the Japanese Classification of Gastric Carcinoma (15th edition) [9] (Tables 9 and 10). In the case of esophagogastric junction squamous cell carcinoma, the stage is used for thoracic esophageal squamous cell carcinoma.

**Table 9:**
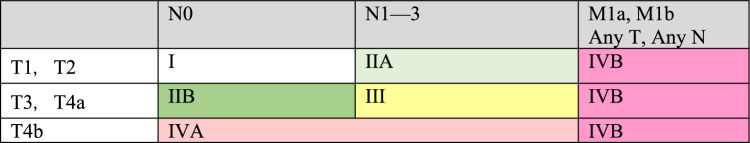
Clinical stage

**Table 10:**
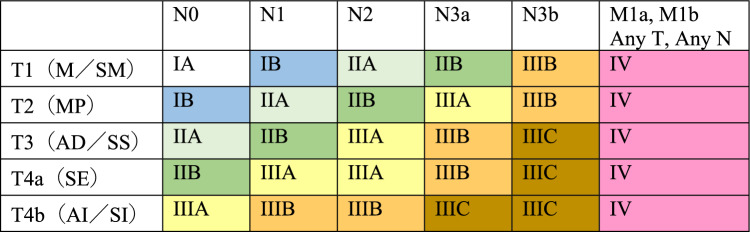
Pathological Stage

### 5.3 Degree of lymph node dissection and residual tumor

#### 5.3.1 Lymph node dissection

**5.3.2 Description of lymph node dissection**

Two-field dissection:

Thoracic and abdominal lymph nodes are dissected. The following lymph nodes must be removed: No. 101, 105, 106rec, 106tbl, 107, 108, 109, 110, 111, 112aoA, 112pul, 1, 2, 3, 7, 8a, 9, 11p (Dissection of No. 106tbl, 111, 8a, and 11p can be omitted).

Three-field dissection:

Cervical ^*Note 1*^, thoracic, and abdominal lymph nodes are dissected. This is equivalent to two-field dissection plus cervical lymph node dissection.

*Note 1:* Cervical lymph node dissection refers to the bilateral resection of No. 101, 102, and 104. Dissection No. 102 can be omitted. Dissection No. 101 can be performed from the thorax.

*Note 2:* The extent of lymph node dissection should be described when typical lymph node dissection is not performed, e.g., thoracic and cervical lymph node dissection (in patients undergoing two-stage esophagectomy without abdominal lymph node dissection).

#### 5.3.3 Definition of extent of lymph node dissection (D)

Based on the location of esophageal cancer, the degree of lymph node dissection is defined as follows.

Cervical esophageal cancer (Ce)

- DX: Degree of lymph node dissection cannot be assessed
- D1: Less than D2 lymph node dissection is performed
- D2: Lymph nodes dissection of 100, 101, 102 mid, 104, 105, and 106rec are performed ^*Note 1, 2*^
- D3: not applicable

*Note 1:* Regarding nodes 105, 106recR, and 106recL, dissection of the nodes that can be resected from the neck is acceptable.

*Note 2:* Although lymph node 102 is a regional node, lymph node dissection without lymph node 102 can be regarded as D2 lymph node dissection.

Thoracic esophageal cancer (Te)

- DX: Extent of lymph node dissection cannot be assessed
- D1: Incomplete dissection of two-field lymph nodes
- D2: Complete dissection of two-field lymph nodes
- D3: Complete dissection of three-field lymph nodes

Esophagogastric junctional cancer (Jz)

- D1: Lymph node dissection of No. 1, 2, 3a, and 7^*Note 1*^
- D1 + : Lymph node dissection of No. 1, 2, 3a, 7, 8a, 9, and 11p^*Note 2*^
- D2: Lymph node dissection of No. 1, 2, 3a, 7, 8a, 9, 11p, 19, 20, and 110^*Note 3*^
- D3: Lymph node dissection of No. 1, 2, 3a, 7, 8a, 9, 11p, 19, 20, 105, 106recL, 106recR, 107, 108, 109L, 109R, 110, 111, 112aoA, and 112pul^*Note 4*^

*Note 1:* The extent of D1 equals nodes with a ≥20% incidence of metastasis in a nationwide prospective study[10] .

*Note 2:* The extent of D1+ equals nodes with a ≥10% incidence of metastasis in a nationwide prospective study[10].

*Note 3:* D2 lymph node dissection is recommended for patients with an esophageal invasion length of 2.1–4.0 cm.

*Note 4:* D3 lymph node dissection is recommended for patients with an esophageal invasion length >4.0 cm.

*Note 5:* When a total gastrectomy is performed, lymph nodes 3b, 4sa, 4sb, 4d, 5, and 6 are added to the extent of the lymph nodes described above.

*Note 6:* In this edition, classification D is based on each surgical procedure. Lymph nodes 4sa and 4sb are excluded from lymph nodes that should be resected because they have a low frequency of metastasis based on a nationwide prospective study[10].

#### 5.3.4 Residual tumor after endoscopic and surgical resection (R)

- RX: Presence of residual tumor cannot be assessed^*Note 5*^
- R0: No residual tumor
- R1: Microscopic residual tumor (resection margin or radial margin)^*Note 2*^
- R2: Macroscopic residual tumor

*Note 1:* Residual tumor should be evaluated for both primary tumor and metastatic lesions.

*Note 2:* The R1 classification includes cases that are highly suspected of having microscopic residual tumors based on frozen sections or exfoliative cytology during the operation.

*Note 3:* When multiple lesions were treated, R classifications are determined separately for each lesion.

*Note 4:* In the case of piecemeal resection, the R classification should be evaluated after restructuring the specimen as much as possible. The pR0 of piecemeal resection is confirmed only when restructuring is possible and non-cancerous tissue is recognized at the resection margins of the restructured specimen.

*Note 5:* RX

1. Non-Cancerous cells cannot be confirmed on the margin because of the crushed or burned effect.
2. Impossible restructuring the specimen because of a piecemeal resection.
3. Suspected residual tumor due to the non-continuous tumor extension in the basal layer of the squamous epithelium.
4. Possible residual tumor in the vertical margin because of intra-ductal spread.
5. Indeterminable residual tumor because of other reasons.

## 6. Handling of endoscopically and surgically resected specimen

### 6.1 Handling of endoscopically resected specimen

The specimen is extended and fixed immediately after resection on a corkboard or polystyrene foam and fixed in a sufficient volume of 10% neutral buffered formalin solution for at least half a day.

### 6.2 Handling of surgically resected specimen (primary tumor)

The resected esophagus should be cut open along the longitudinal line on the opposite side of the lesion. Next, it should be gently stretched longitudinally and fixed such that the length of the specimen becomes similar to its size in vivo. Photographic recordings are necessary for both fresh and fixed specimens.

The specimen is extended after resection on a corkboard or polystyrene foam and fixed in a sufficient volume of 10% neutral buffered formalin solution. The recommended fixation time is < 72 h.

### 6.3 Sectioning of resected specimen

#### 6.3.1 Preparation of resected specimens for sectioning

The formalin-fixed specimen should be treated with an iodine solution to confirm the unstained area and accurately recognize and record the macroscopic findings. This is important for the treatment of superficial carcinomas. Rinsing the sample with tap water for at least 30 min for endoscopically resected specimens and at least 1 h for surgically resected specimens will provide good staining conditions. To increase the contrast between the stained and unstained areas, the sample should be treated with a relatively low concentration (0.1–0.5%) of iodine solution for a long time.

#### 6.3.2 Rule of sectioning for endoscopically resected specimen (Fig. 27)

**Fig. 27:**
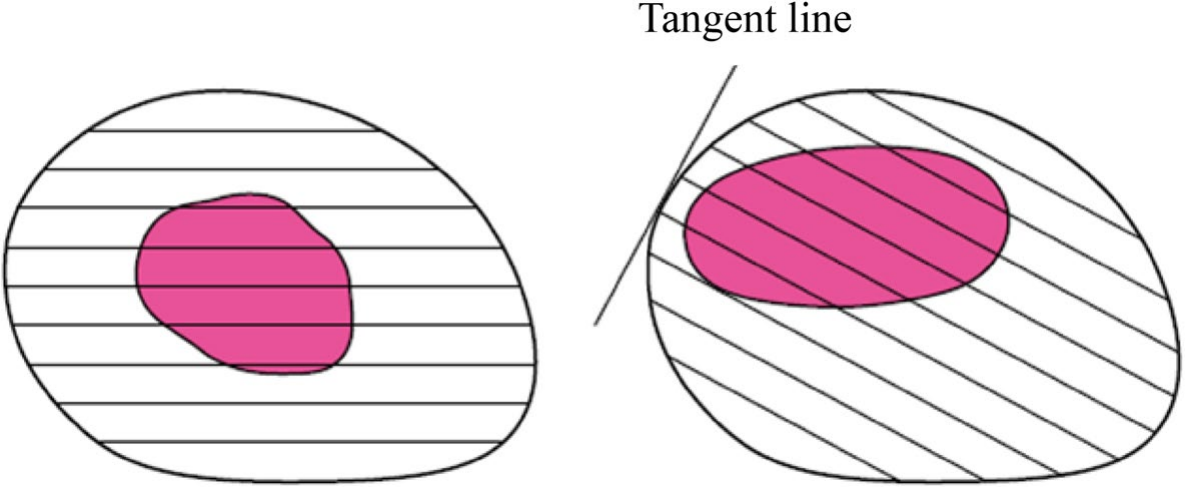
How to cut endoscopically resected specimens

Cutting lines are defined as crossing lines at right angles to the tangent line at the resection margin closest to the tumor, and a whole resected specimen is cut into slices each at 2–3 mm thick.

#### 6.3.3 Rule of sectioning for surgically resected specimen (Fig. 28)

**Fig. 28:**
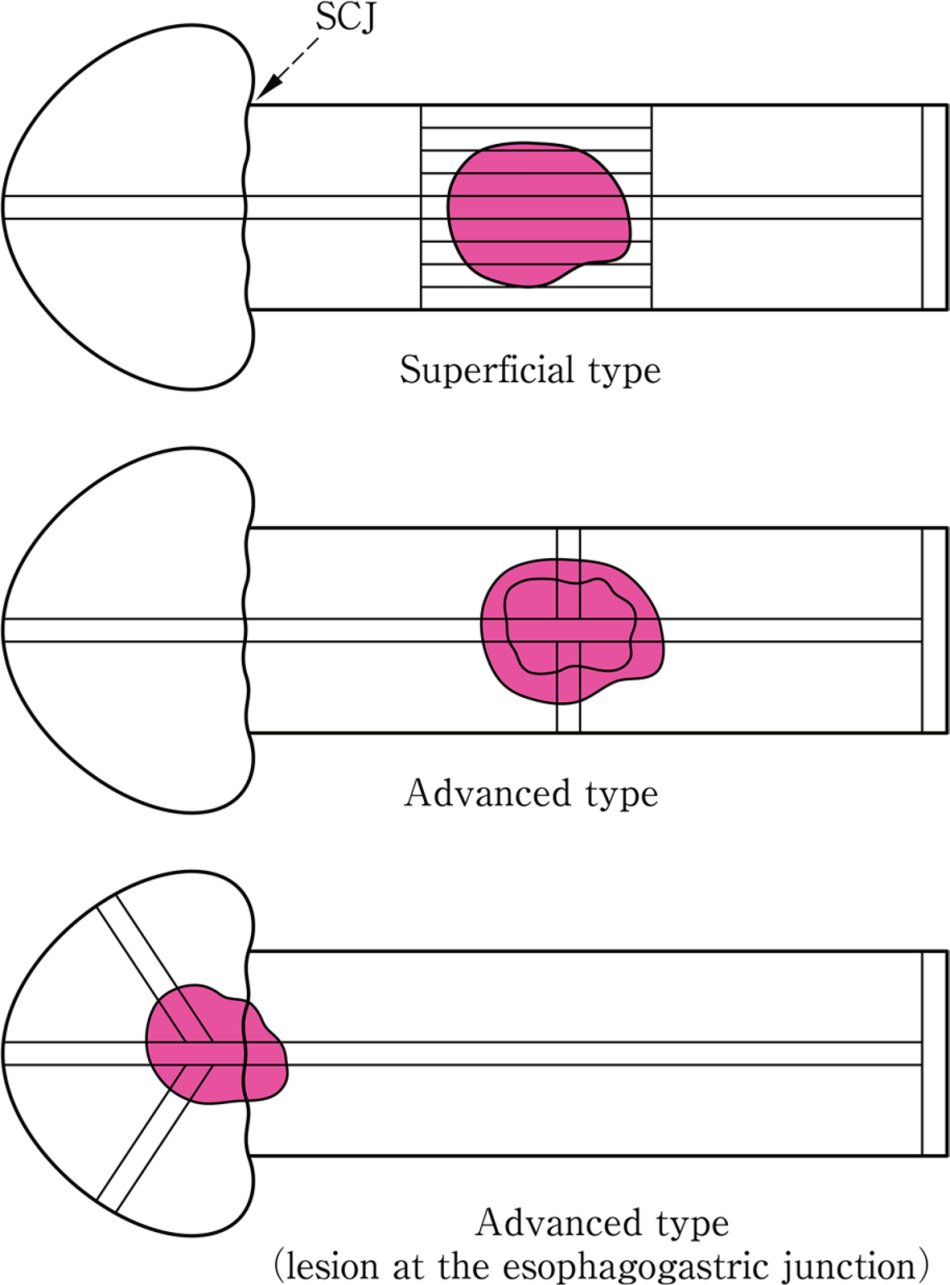
How to cut surgically resected specimens

The resected specimen should be cut parallel to the long axis of the esophagus. Whole-step sections should be prepared for superficial cancer. A representative section of an advanced tumor at the site of the deepest invasion, parallel or perpendicular to the esophagus should be blocked and used for microscopic examination. The entire tumor should be cut into sections for macroscopic observation, and the adequate sections must be chosen for microscopic examination. The section of the proximal margin must be made parallel or perpendicular based on macroscopic findings. Schemas or photographs of the cut section sites should be preserved.

## Data Availability

Data sharing is not applicable to this article as no datasets were generated or analyzed during the current study.
